# SAHRANG: Subarachnoid Hemorrhage Recovery and Galantamine: A Pilot Multicenter Randomized Placebo-Controlled Trial

**DOI:** 10.1007/s12028-025-02349-3

**Published:** 2025-08-28

**Authors:** Bosco Seong Kyu Yang, Jude P. J. Savarraj, Elena Moreno, Kevin E. Immanuel, Georgene Hergenroeder, Glenda Torres, Jung Hwan Kim, Sophie Samuel, Claudia Pedroza, James C. Grotta, Andrew Barreto, H. Alex Choi

**Affiliations:** 1 Departent of Neurosurgery, McGovern Medical School, The University of Texas Health Science Center at Houston, Houston, TX USA; 2 Department of Neurology, Houston Methodist Hospital, Houston, TX USA; 3 Department of Pediatrics, McGovern Medical School, The University of Texas Health Science Center at Houston, Houston, TX USA; 4 Department of Neurology, McGovern Medical School, The University of Texas Health Science Center at Houston, Houston, TX USA

**Keywords:** Acute brain injury, Inflammation, Subarachnoid hemorrhage, Galantamine, Cholinesterase inhibitor

## Abstract

**Background:**

Subarachnoid hemorrhage (SAH) causes life-long neurologic dysfunction. Peripheral inflammatory processes as a reaction to brain injury have been shown to worsen outcomes after SAH. Galantamine has been shown to reduce proinflammatory microglial activities and improve synaptic connections. We hypothesize that galantamine treatment after SAH mitigates inflammation-mediated neuronal injury and improve outcomes. We conducted a pilot clinical trial to examine the tolerability and safety of galantamine in patients with SAH.

**Methods:**

This prospective, multicenter, double-masked, randomized, placebo-controlled study contiguously screened and enrolled adult patients presenting with aneurysmal SAH with a Fisher grade of 3 within 72 h of symptom onset. A total of 60 patients were enrolled with a 1:1 ratio to two treatment arms. The first 30 patients were randomly assigned to receive galantamine at 8 mg every 12 h or a placebo, and the other 30 patients to were randomly assigned to receive either galantamine at 12 mg every 12 h or a placebo. All medications were started within 36 h after securing the aneurysm and continued for 90 days. Primary outcomes—tolerability as assessed by the number of patients who stop study medication due to adverse events associated with the study drug and mortality due to the study drug—were assessed at 90 days.

**Results:**

There were no differences in tolerability and safety between the two groups. Bradycardia was the most common adverse event (37%), followed by clinical seizure (3%) and skin rash (3%). One study participant in the galantamine group discontinued medication due to a skin rash, and another study participant from the placebo group discontinued due to nausea (*p* = 0.92). Mortality did not differ between the two groups. At 90 days, one study participant from the galantamine group and four study participants from the placebo group died (*p* = 0.34).

**Conclusions:**

Galantamine was as tolerable and safe as a placebo based on discontinuation rates and mortality in patients with SAH when administered to patients with SAH during the early and subacute stages of the disease.

**Supplementary Information:**

The online version contains supplementary material available at 10.1007/s12028-025-02349-3.

## Introduction

Subarachnoid hemorrhage (SAH) is caused by sudden rupture of a cerebral aneurysm. Although SAH represents only 10% of strokes, mortality is high (up to 35%), and morbidity is significant because patients are often young and left with permanent cognitive and functional deficits [1]. The exact mechanism linking the initial insult to eventual comorbidity remains elusive, focusing scientific attention on molecular and cellular pathophysiology [2].

Uncontrolled inflammation is an essential component of this mechanism, supported by both clinical and animal studies [3, 4]. Recent preclinical studies have suggested the importance of nicotinic α7 receptors in regulating inflammation by demonstrating that stimulation of the receptors reduces the levels of inflammatory cytokines [5]. Galantamine is an acetylcholinesterase inhibitor (AChEi) with the ability to modulate α7 nicotinic receptors allosterically and was approved in 2001 by the US Food and Drug Administration for the treatment of mild to moderate Alzheimer disease [6]. Galantamine has been found to be effective in improving cognitive outcomes in vascular dementia and psychiatric and motor functions in Parkinson disease [7, 8]. Among available AChEis, galantamine has shown the strongest long-term treatment effect on cognitive functions [9]. Recent studies suggest the anti-inflammatory property of galantamine has beneficial pleiotropic effects [10]. Preclinical studies show that galantamine decreases proinflammatory cytokines, such as interleukin 1β (IL-1β), interleukin 6 (IL-6), and tumor necrosis factor α (TNF-α), resulting in better hippocampal neuronal survival and improved functional outcome in preclinical studies [11].

In addition to these conventional roles of galantamine in neurodegenerative diseases, more recent studies have suggested that galantamine can ameliorate neurological symptoms from acute brain injury. Galantamine has been shown to improve episodic memory, alleviate depressive symptoms in patients with mild traumatic brain injury, and improve chronic aphasia due to remote ischemic stroke [12, 13]. The effect of galantamine in patients with SAH has not been studied. We conducted a pilot clinical trial of galantamine in patients with SAH. We examined the tolerability and safety of galantamine given within 36 h of SAH onset to up to 90 days after hospitalization, its preliminary efficacy on functional and cognitive outcomes, and its influence on proinflammatory cytokines. This study has been registered at ClinicalTrials.gov under registration no. NCT02872857.

## Methods

### Study Design

This was a prospective, multicenter, double-masked, randomized, placebo-controlled study in adult patients with acute aneurysmal SAH. The aim was to investigate safety, tolerability, and preliminary efficacy of galantamine in improving long-term outcomes of patients with acute SAH. The study spanned over 36 months from 2016 September to 2019 July. Patients were followed for 90 days after enrollment. As primary safety outcomes, adverse events and mortality from study products were monitored. An interim analysis was conducted after 30 study participants were enrolled to confirm tolerability and safety. This study’s protocol, informed consent documents, and any subsequent modifications were reviewed and approved by the University of Texas Health Science Center at Houston institutional review board and ethics committee. The study met all requirements, including protection of human study participants and a data safety and monitoring board (DSMB), and followed Consolidated Standards of Reporting Trials guidelines for randomized pilot trials [14].

### Study Participants

Consecutive study participants admitted and hospitalized at the Memorial Herman Hospital System and the Methodist Hospital System in Houston, Texas, were screened for inclusion if they met the following criteria: had spontaneous SAH, presentation to hospital within 72 h of symptoms, aged 18–75 years, had Fisher grade 3 hemorrhage (thick subarachnoid clot) on initial computed tomography scan, Hunt and Hess grade 1–5 at the time of randomization, presence of a cerebral aneurysm on computed tomography angiogram or angiogram for which clipping or coiling is possible, and having the ability to receive medication within 36 h of securing an aneurysm. Study participants were excluded if they had SAH due to other causes (including trauma, arteriovenous malformation, mycotic aneurysms, moyamoya), renal disease (defined by a creatinine clearance of less than 9 ml/min), history of severe hepatic impairment (Child–Pugh score of 10–15), history of chronic obstructive pulmonary disease or asthma, comorbid conditions likely to complicate therapy (including clinically significant arrhythmia, acquired immunodeficiency syndrome, autoimmune disease and malignancy), expected mortality within 72 h, inability to complete long-term follow-up, severe functional disabilities before hospital admission for SAH (defined by preadmission modified Rankin Score [mRS] > 1), preexisting systemic diseases that would impact long-term outcomes (including stroke, myocardial infarction, pulmonary disease requiring home oxygen, chronic renal failure necessitating hemodialysis, and malignancy), history of dementia, documented neurologic and psychiatric disorders, or if they were prisoners or pregnant women. The enrollment was restricted to patients with Fisher grade 3 to maximize the chance of enrollment. Previous studies suggest 68% of study participants with SAH present with a Fisher grade of 3 [15]. The restrictive inclusion criterion also intended to ensure that cognitive benefits of the intervention could be observed, if any exists, given that more severe SAH is associated with a higher incidence of cognitive complications [16]. Upon enrollment, demographic and clinical information were collected. This study was designed as a pilot study for the planning of larger future studies, so it aimed to enroll a sample size deemed feasible during the study period, which was 60 study participants.

### Randomization

To balance the severity of SAH between the control and treatment groups, we used a 1:1 ratio permuted block randomization. The Hunt and Hess Score (HH) is a score based on neurologic examination that represents the severity of SAH and is a strong predictor of mortality after SAH [17]. Patients who present with HH 4 or 5 are at a much higher risk of dying compared with patients who present with HH 1, 2, or 3. To account for this, we permuted blocks of two to four, stratifying based on admission HH (HH 2 and 3 versus HH 4) to ensure that groups are balanced with respect to clinical severity. Randomization was performed by research pharmacy.

### Intervention

All patients were cared for in the neuroscience intensive care unit by experienced neurosurgeons and neurointensivists and received standard of care per American Heart Association guidelines for treating patients with SAH.

In the first phase of the trial, 30 patients were randomly assigned to receive galantamine at 8 mg every 12 h or a placebo and were started within 36 h after securing the aneurysm. Galantamine and placebo were administered orally and were indistinguishable from each other. At the end of the first phase, an interim analysis for tolerability and safety was performed. After the tolerability and safety were confirmed, another 30 patients were randomly assigned to receive either galantamine at 12 mg every 12 h or a placebo. For dosage adjustments, if more than nine antiemetic agents were given within 48 h or a serious adverse event (SAE) occurred, the principal investigator, the treating physician, and the treating clinical pharmacist convened and determined whether the dosage should be decreased. The dosage adjustment criteria are based on our retrospective examination of 141 patients admitted with SAH. We found that 80% of the patients received antiemetic medications, with a median dose requirement of four doses (interquartile range [IQR] 2–9 doses). Thus, the 75% quartile of nine doses was used as a screening tool to detect possible intolerability of galantamine. If it was decided that the dose should be decreased, the dose was decreased by 4 mg from 8 to 4 mg or from 12 to 8 mg. This occurred while all the associated personnel were masked to treatment allocation. The principal investigator asked the research pharmacy to decrease the dose, and the dose was decreased, regardless of whether the treatment allocation was galantamine or placebo. Once a tolerable dose was found, the study participant was maintained on the dose for a total of six doses, and an attempt to increase the dose by 4 mg per dose was made until we reached the target dosage of 8 mg twice a day in the first phase or 12 mg twice a day in the second phase. If two attempts were made to reach the target dose but tolerability issues persisted, the study participant remained on the tolerable dose until the end of the 90-day period.

### Safety Outcomes

Throughout the study, patients were closely monitored for potential side effects of the medication. The monitored adverse effects included arrhythmias, gastric ulcers, urinary retentions, seizures, and nausea and vomiting. Adverse outcomes were examined with the Common Terminology Criteria for Adverse Events Version 4.0. When an adverse event occurred, causality was examined. All SAEs meeting grade 4 criteria were communicated to the DSMB. All SAEs that were unexpected and related to the intervention were reported to the DSMB within 7 calendar days of the determination by telephone, email, or fax. All other SAEs were collected and submitted with the report to the DSMB. Meetings with DSMB were held before trial initiation, and subsequent meetings were held after the first 30 study participants completed the 90-day mRS.

### Efficacy Outcomes

The primary efficacy outcome was mRS at 90 days after ictus. mRS was measured at the time of discharge, 30, 60, and 90 days after ictus. The Montreal Cognitive Assessment (MoCA) score and the EuroQOL 5-level EQ 5D (EQ5D5L) self-rated health status at 90 days were secondary outcomes for cognitive functions and quality of life (QOL), respectively. Cognitive outcomes were measured at 30, 60, and 90 days after ictus, and a baseline cognitive status was measured with MoCA, either at discharge or 14 days after admission. MoCA scores of 26–30 show normal cognitive function, whereas scores 0–17 indicate severe cognitive impairment. The EQ5D5L participant responses rate 5 dimensions by 5 levels of severity with higher levels representing more severe problems.

### Ancillary Outcomes

We assessed the levels of proinflammatory markers (central and systemic inflammation) across both arms as ancillary outcomes. Serum and cerebrospinal fluid (CSF) samples were collected at eight specified intervals depending on the type of samples: within 24 h of admission (T1), 24–48 h (T3), days 3–5 (T4), days 6–8 (T5), more than 8 days but prior to discharge (T6), and at 30 (TM30), 60 (TM60), and 90 (TM90) days after ictus. In total, 456 blood samples were obtained from 60 study participants, and 188 CSF samples were obtained from 55 study participants.

For plasma samples, blood was withdrawn from existing lines or by venipuncture and collected into two pink top K2 EDTA 6-ml Vacutainers per time point (36 ml). The samples were placed on ice and were centrifuged at 1,459 × g for 10 min at 4 °C. The supernatant solution was removed and centrifuged at 1,459 × g for 10 min at 4 °C to generate platelet-poor plasma. Plasma was divided into aliquots and frozen at − 80 °C until needed. For CSF samples, sample collection required patients to maintain an admitted state, so they were collected at only five time points before their discharge. A fresh 1–5-ml sample of CSF was obtained from the ventriculostomy burette-type drain receptacle at each of the five time points. The receptacle was emptied into the collection bag to start with an empty receptacle, and several drops of CSF were allowed to drip into the chamber. The drain was reclamped; the chamber port was cleaned with chlorhexidine as per hospital specifications, and the CSF sample was drawn from the port with a sterile syringe. CSF was centrifuged (4 °C, 1,459 × g for 10 min), portioned into aliquots, and frozen at − 80 °C; any residual sample was frozen at − 80 °C for future study. The CSF pellet was covered in a 10% dimethyl sulfoxide solution (e.g., 100 µl CSF plus 11 µl dimethyl sulfoxide) and frozen at − 80 °C and transferred to − 145 °C after 24 h when possible.

We measured the levels of inflammatory cytokines using a 17-plex premixed immunological multiplex assay (HCYTMAG-60 K-PX38; MilliporeSigma, Billerica, MA). Analyzed cytokines included Eotaxin/CCL11, PDGFABBB, MCP1, GCSF, sCD40L, MIP1a, GMCSF, IL-1RA, MIP1b, IFNg, IL-6, RANTES, IL-10, IL-8, TNF-α, PDGFAA, and IP10. All experiments were performed according to the manufacturer’s protocol. The levels of all markers are reported as pg/ml.

### Statistical Analysis

A prespecified safety interim analysis was performed after 30 patients were enrolled and before proceeding to the second phase of the study. Safety outcomes were dose tolerability defined as either the patient dropping out of the study because of side effects, the physician discontinuing the medication because of side effects, or the occurrence of SAEs possibly related to the medication. Both outcomes were analyzed with Bayesian logistic models including treatment group and HH grade (stratifying variable) as covariates. A neutral prior center at an odds ratio of 1.0 with a 95% confidence interval (CI) of 0.33–3.0 (normal distribution with a mean of 0 and standard deviation of 0.57 in the log odds ratio scale) was used for the treatment effect. All other priors were neutral and weakly informative. The decision rule for decreasing the starting dose would be met if either (1) the galantamine group had more than a 30% rate of intolerability or (2) there was a > 90% posterior probability of increased rate of patients with at least one in SAE in the galantamine arm compared with the placebo arm. As for efficacy outcomes, because this study was a pilot and feasibility trial, no sample size calculation or power analysis was conducted.

Continuous variables are reported as means ± standard errors. Dichotomized and categorical variables are reported as proportions, whereas Fisher’s exact test and Wilcoxon rank-sum tests were used accordingly to test statistical significance. Incidence of primary outcomes, tolerability and mortality, was compared between the two treatment groups with Fisher’s exact test. For efficacy outcomes at each time point, distributions of mRS were compared with the *χ*^2^ test with continuity correction, and MoCA scores, EQ5D5L VAS, and EQ5D5L indices were compared using the Wilcoxon rank-sum test. Changes in functional, cognitive, and QOL outcomes over time between assessments were calculated as a difference in outcome measures between contiguous time points. Two post hoc analyses were conducted to analyze the effects of galantamine on EQ5D5L VAS. First, the relationship between treatment group assignment and changes in individual components of EQ5D5L were analyzed, adjusting for age, sex, ethnicity, HH grade, and disease stages. Second, the interaction between treatment groups and disease stages was analyzed by adding an interaction term. Both models used a fixed effect linear regression model to account for between–study participant variations. Ancillary outcomes, including incidence of delayed complications and plasma levels of inflammatory cytokines at different time points were also compared using Fisher’s exact test and Wilcoxon rank-sum test, accordingly. A conventional threshold of 0.05 was used for the *p* value to define statistical significance. All statistical analyses were performed using open-source software packages in R (v4.3.3).

## Results

**Fig. 1:**
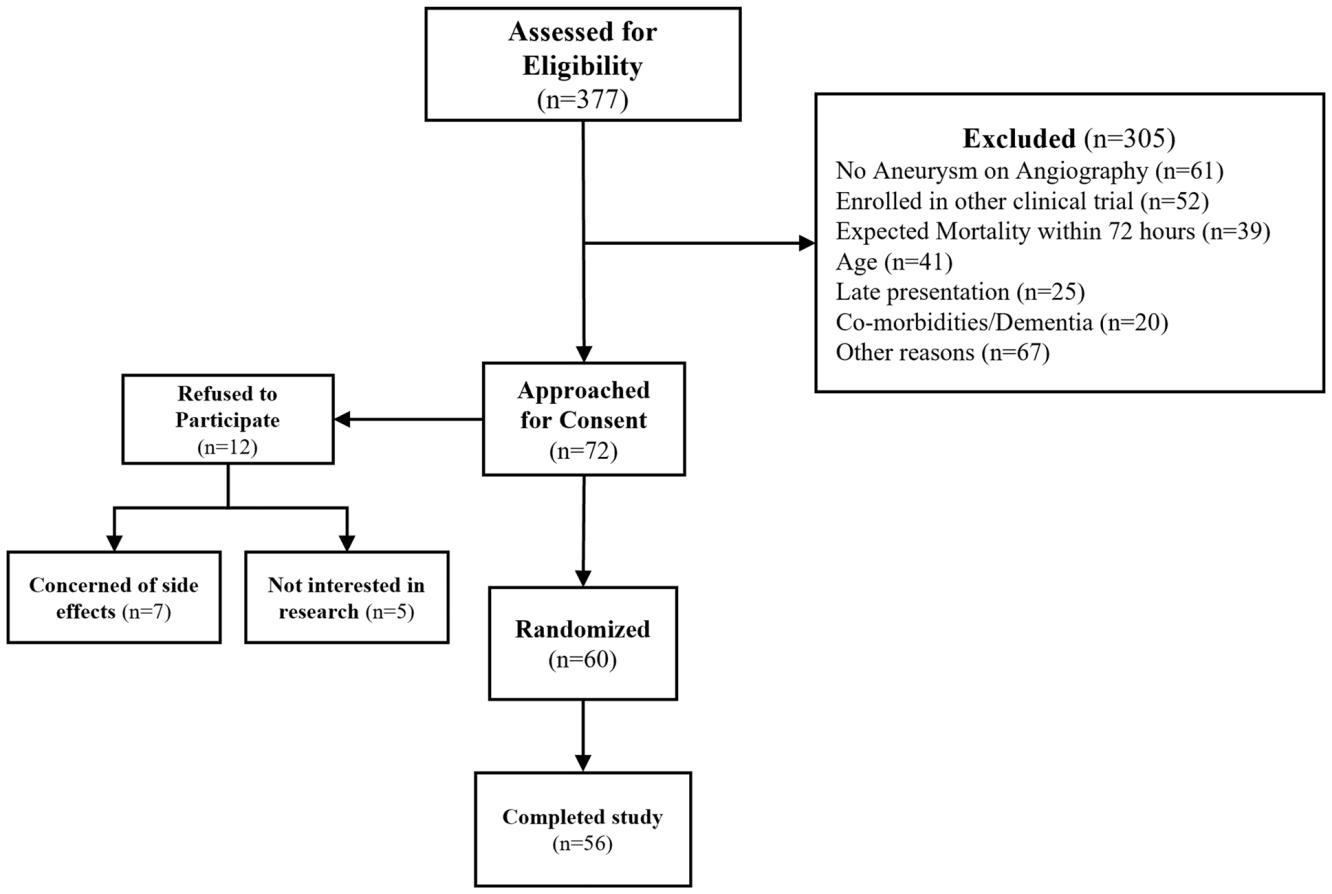
Flow diagram showing screening, exclusion, consent, randomization, and follow-up design

From October 2016 to April 2019, we consecutively screened 377 patients with SAH for eligibility (Fig. 1). A total of 315 patients were excluded, including 19% who did not have an aneurysm, 16% who were enrolled in other trials, 13% who were older than the inclusion criterion, and 12% who were expected to die within 72 h. A total of 72 study participants were approached for consent, and 60 study participants were randomly assigned to either the galantamine or the placebo group (Fig. 1). Two study participants from the galantamine group and another two study participants from the placebo group were lost to follow-up; however, all four study participants were confirmed to have returned to neurological baseline and were back at work by 2 months. The last-observation-carried-forward-method was used to impute the 90-day outcomes for study participants who missed the in-between follow-ups.

The average age of the study participants was 51 years, and 70% were female. A total of 80% of the participants had a history of hypertension (Table 1). There were no differences in age, sex, ethnicity, and race across the two groups (Table 2). Because the study participants were randomized to the group based on clinical severity at admission, there were no differences in the HH or the World Federation of Neurosurgical Societies Scale score. There were no differences in the location of the aneurysms or modes of treatment to secure aneurysms across the two groups (Table 2).

**Table 1:** Baseline characteristics of overall population

	Total (n = 60)
Age (mean, sd)	51 (12)
Female (n, %)	42 (70%)
Hispanic (n, %)	33(55)
BMI (mean kg/m2(sd))	28(6)
OSH transfer (n, %)	46(77)
Hypertension	48(80)
Hyperlipidemia (n, %)	14(23)
Tobacco, (n, %)	30(50)
Cannabis, (n, %)	6(10)

*BMI*: Body mass index

*OSH*: Outside hospital transfer

**Table 2:** Baseline characteristics stratified by treatment arms

		Galantamine	Placebo	*p* values
Age		48.3 (12.6)	54.2 (10.4)	0.12
Sex (n, %)	Female	19 (63.3)	23 (76.7)	0.4
Ethnicity	Hispanics	17 (56.7)	16 (53.3)	0.9
Race (original)	Black	8 (26.7)	4 (13.3)	0.33
	White	20 (66.7)	25 (83.3)	
	Other	2 (6.6.3)	1 (3.3)	
HH at randomization	2	7 (23.3)	3 (10.0)	0.48
	3	9 (30.0)	12 (40.0)	
	4	10 (33.3)	9 (30.0)	
	5	4 (13.3)	6 (20.0)	
WFNS grade on arrival	1	9 (30.0)	3 (10.0)	0.18
	2	6 (20.0)	10 (33.3)	
	4	4 (13.3)	7 (23.3)	
	5	11 (36.7)	10 (33.3)	
Worst WFNS grade in 24 h	1	4 (13.3)	1 (3.3)	0.34
	2	5 (16.7)	6 (20.0)	
	4	4 (13.3)	8 (26.7)	
	5	17 (56.7)	15 (50.0)	
GCS		8.0 (5.0–13.8)	8.5 (5.2–13.0)	0.8
Aneurysm location	LICA	2 (6.7)	2 (6.7)	0.56
	BA	0 (0.0)	1 (3.3)	
	RVA	2 (6.7)	0 (0.0)	
	LPICA	0 (0.0)	2 (6.7)	
	RPICA	2 (6.7)	0 (0.0)	
	Pericallosal	1 (3.3)	0 (0.0)	
	RSCA	1 (3.3)	0 (0.0)	
	RICA	1 (3.3)	2 (6.7)	
	L.ant.choroidal	0 (0.0)	1 (3.3)	
	LACA	0 (0.0)	1 (3.3)	
	LMCA	3 (10.0)	3 (10.0)	
	RMCA	1 (3.3)	3 (10.0)	
	ACom	8 (26.7)	7 (23.3)	
	Lpcom	4 (13.3)	3 (10.0)	
	Rpcom	5 (16.7)	5 (16.7)	
Clipping	No	21 (70.0)	18 (60.0)	0.59
	Yes	9 (30.0)	12 (40.0)	
Coiling	No	9 (30.0)	11 (36.7)	0.78
	Yes	21 (70.0)	19 (63.3)	
Parenchymal hematoma	No	11 (68.8)	12 (92.3)	0.18
	Yes	5 (31.2)	1 (7.7)	

### Primary Tolerability and Safety Outcomes

An interim analysis revealed no statistical difference in primary safety outcomes between the two treatment arms. In both groups, at least one study participant with an SAE was observed, and the overall rates of intolerability were < 30% in both groups. The posterior probability of an increased rate of SAEs in the group with a higher observed incidence rate of SAEs compared with the other group was 72%, which was lower than the preset stopping rule.

The final analysis showed no significant differences in the incidence of any adverse events across the two groups. A total of 135 and 145 adverse events were observed in 29 and 30 patients in the galantamine and the placebo groups, respectively, with the relative risk of 1.05 (95% CI 0.83–1.33) comparing the galantamine group to the placebo group (Table 3). The risk of any SAE, with the relative risk of 1.33 (95% CI 0.8–2.22) was not different between the groups (Table 3). We found no differences in the incidence of adverse events, including death, clinical seizures, bradycardia, tachycardia, pneumonia, hyponatremia, and anemia. The galantamine group had fewer incidences of pulmonary edema and pulmonary embolism, but the difference did not reach significance (*p* > 0.05) (Table 3). The two groups also were not different in the risk of any adverse events or any SAE directly attributable to the investigational product (Table 4).

**Table 3:** Incidence of adverse events stratified by treatment arms

All adverse events	Galantamine (n = 30)		Mean (SD)	Placebo (n = 30)		Mean (SD)	RR (95% CI)	*p* value
	Total events	Patients (n)		Total events	Patients (n)			
Any adverse event	135	29	4.5 (2.5)	145	30	4.8 (2.7)	1.05 (0.83–1.33)	0.7
Any serious adverse event	26	17	0.87 (0.97)	36	17	1.2 (1.5)	1.33 (0.80–2.22)	0.27
Adverse event resulting in discontinuation of IP (investigational product)	1	1	0.03 (0.18)	1	1	0.03 (0.18)	0.86 (0.05–14.06)	0.92
Death during hospital stay	0	0	–	1	1	0.03 (0.18)	–	–
Death at 90 days	1	1	0.03 (0.18)	4	4	0.13 (0.35)	3.03 (0.32–28.83)	0.34
Distribution of adverse events								
Delayed cerebral ischemia	12	10	0.4 (0.6)	15	12	0.5 (0.7)	1.30 (0.61–2.79)	0.5
Clinical seizure	2	1	0.07 (0.37)	2	2	0.07 (0.25)	1.00 (0.14–7.10)	> 0.99
Skin rash	2	2	0.07 (0.25)	1	1	0.03 (0.18)	0.53 (0.05–5.81)	0.6
Bradycardia	11	11	0.37 (0.49)	12	12	0.4 (0.5)	1.05 (0.46–2.39)	0.91
Tachycardia	8	8	0.27 (0.45)	5	5	0.17 (0.38)	0.60 (0.2–1.85)	0.37
Urinary retention	2	2	0.07 (0.25)	5	5	0.17 (0.38)	2.33 (0.45–12.16)	0.32
Urinary tract infection	9	9	0.3 (0.47)	6	6	0.2 (0.41)	0.69 (0.25–1.94)	0.48
Pneumonia (any)	11	9	0.37 (0.61)	9	9	0.3 (0.47)	0.84 (0.35–2.02)	0.7
Pulmonary edema	1	1	0.03 (0.18)	5	5	0.17 (0.38)	4.88 (0.56–42.29)	0.15
Angio complications (any)	3	2	0.1 (0.4)	1	1	0.03 (0.18)	0.36 (0.04–3.46)	0.38
Surgical complications (any)	1	1	0.03 (0.18)	0	0	–	–	
Nausea	0	0	–	2	2	0.07 (0.25)	–	
Hyponatremia	12	12	0.4 (0.5)	12	11	0.4 (0.56)	1.03 (0.46–2.3)	0.94
Hypernatremia	7	7	0.23 (0.43)	11	11	0.37 (0.49)	1.47 (0.57–3.83)	0.43
Cerebral edema	3	3	0.1 (0.31)	3	3	0.1 (0.31)	1.00 (0.20–4.94)	> 0.99
Anemia	13	13	0.43 (0.50)	12	12	0.4 (0.5)	0.91 (0.41–2.00)	0.81
Pulmonary embolism	2	2	0.07 (0.25)	8	8	0.27 (0.45)	3.49 (0.73–16.73)	0.12
Atrial fibrillation (A–fib)	0	0	–	2	2	0.07 (0.25)	–	
Other	36	19	1.2 (1.32)	34	16	1.13 (1.36)	0.89 (0.56–1.44)	0.65

**Table 4:** Possibility of Adverse Events related to IP

Possible or definitely related to the I.P	Galantamine (n = 30)		Mean (sd)	Placebo (n = 30)		Mean (sd)	RR (95% CI)	*p* value
	Total events	Patients (n)		Total events	Patients (n)			
Any adverse event	15	12	0.5 (0.7)	19	17	0.6 (0.6)	1.23 (0.62–2.43)	0.55
Any serious adverse event	2	2	0.07 (0.25)	0	0	–	–	
Adverse event resulting in discontinuation of IP	1	1	0.03 (0.18)	1	1	0.03 (0.18)	0.86 (0.05–14.06)	0.92
Death during hospital stay	0	0	–	0	0	–	–	
Distribution of adverse events								
Delayed cerebral ischemia	0			0				
Clinical seizure	2	1	0.07 (0.37)	0	0	–	–	
Skin Rash	1	1	0.03 (0.18)	0	0	–	–	
Bradycardia	11	11	0.37 (0.49)	12	12	0.4 (0.5)	1.05 (0.46–2.39)	0.91
Tachycardia	0			0				
Urinary retention	0	0	–	5	5	0.17 (0.38)	–	
UTI	0			0				
Pneumonia (any)	0			0				
Pulmonary edema	0			0				
Angio complications (any)	0			0				
Surgical complications (any)	0			0				
Nausea	0	0	–	2	2	0.07 (0.25)	–	
Hyponatremia	0			0				
Hypernatremia	0			0				
Cerebral edema	0			0				
Anemia	0			0				
Pulmonary embolism	0			0				
Atrial fibrillation (A–fib)	0			0				
Other	1	1	0.03 (0.18)	0	0	–	–	

The two treatment arms were not different in terms of mortality. At the time of discharge, one patient from the placebo arm had died. At 90 days after ictus, one patient in the galantamine group and four patients in the placebo group had died. The differences in mortality at both time points did not reach statistical significance (*p* > 0.05) (Table 3).

### Secondary Efficacy Outcomes

**Fig. 2:**
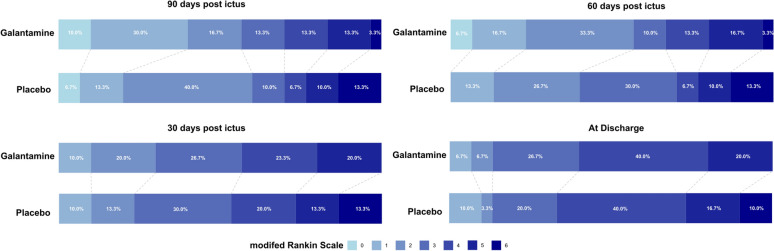
Distribution of the modified Rankin Scale at different time points. Modified Rankin Scale (mRS) measured at 90 days after ictus did not differ between patients with SAH on either galantamine (n = 30) or placebo (n = 30). Higher proportion of patients in the galantamine group achieved mRS of 0 or 1, compared to the placebo group, but the difference was not statistically significant. Same observations were made at other time points (at discharge, 30, and 60 days after ictus)

**Fig. 3:**
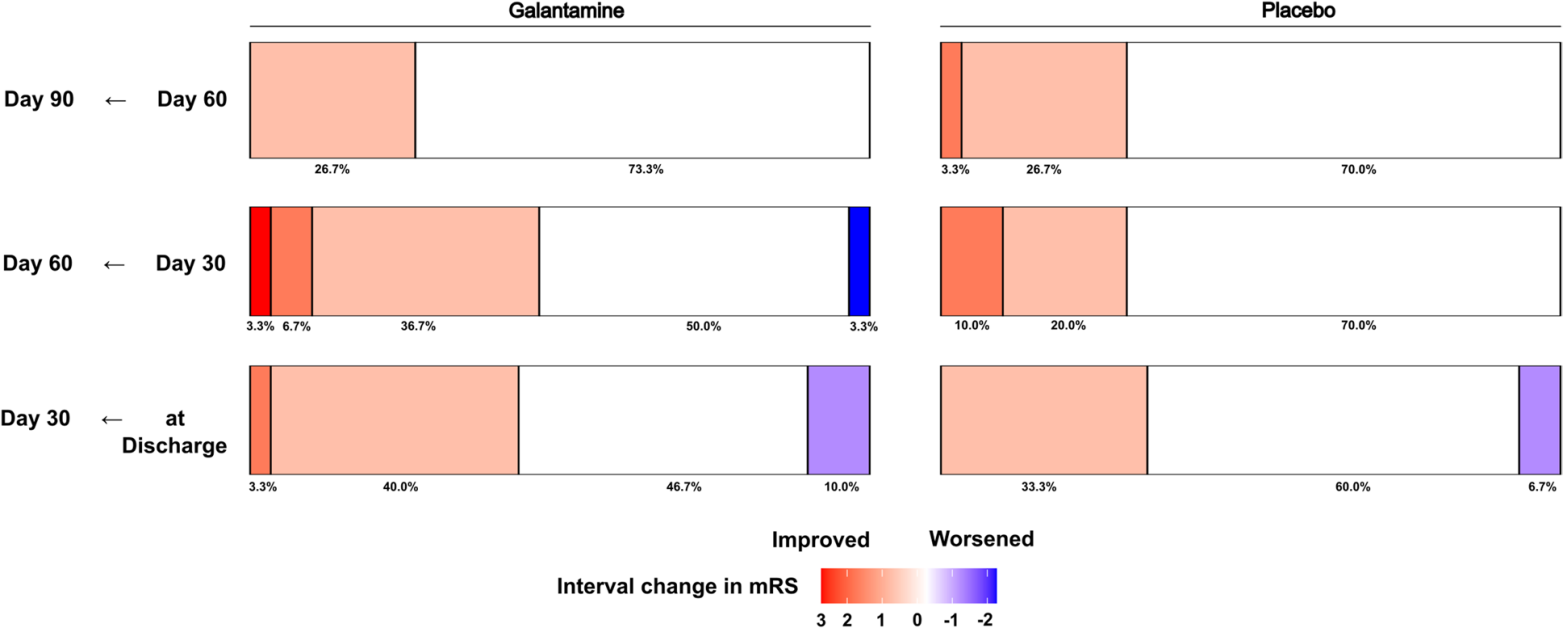
Comparison of the changes in the modified Rankin Scale between time points. Functional improvement, measured with the temporal difference in modified Rankin Scale, was observed in a larger portion of the patients in the galantamine group, compared to the placebo group. The differences were especially large during the earlier stage, within 60 days after ictus. However, all differences were not statistically significant

 At 90 days after ictus, 17 (57%) patients in the galantamine arm and 18 (60%) patients in the placebo arm achieved mRS < 3 with a median mRS of 2 for both groups. The difference did not reach statistical significance (*p* > 0.05) (Fig. 2, Table 5). A higher proportion of patients achieved mRS of 0 or 1 in the galantamine group (40%) in comparison to the placebo group (20%), but the difference lacked statistical significance. Distributions of mRS were not different between the two intervention arms at other time points—at discharge, 30 days, and 60 days after ictus (Fig. 2). Examining differences in changes over time, we found that although the galantamine group consistently showed more improvement in a higher proportion of patients achieving improvement in their functional status during an early phase, we were underpowered to achieve statistical significance. Between hospital discharge and 30 days after ictus, 43.3% and 33.3% of the patients showed improvement of mRS in the galantamine and the placebo groups, respectively (Fig. 3). Similarly, between 30 and 60 days after ictus, functional improvement was observed in 46.7% and 30% of the patients in the two intervention arms, respectively (Fig. 3). Despite the difference in the proportion of patients who achieved functional improvements, the magnitude of improvement did not differ between the two arms. For the first, second, and third 30-day intervals, the galantamine group’s mRS improved by 0.37, 0.53, and 0.27 on average, whereas the placebo group’s mRS improved by 0.27, 0.4, and 0.3 (Supplementary Fig. 1). Yet, the differences in the proportion and magnitude of functional improvements did not reach statistical significance (*p* > 0.05) (Fig. 3, Supplementary Fig. 1).

**Table 5:** Functional Outcomes, cognitive Outcomes and quality of life

	Galantamine	Placebo	*p* value
modified Rankin scale median(IQR)			
30 day	3(2–4)	3(3–4.7)	0.48
60 day	2(2–4)	3(2–4)	0.28
90 day	2(1–4)	2(2–4)	0.37
Montreal cognitive outcomes mean(SD)			
30 day (n = 51), mean(SD)	11(10.4)	13.2(9.5)	0.5
60 day (n = 48), mean(SD)	12.7(10.9)	12.7(9.8)	0.6
90 day (n = 48), mean(SD)	14(11.5)	15.8(10.1)	0.6
EQ5D5L VAS value mean(SD)			
30 day (n = 53), mean(SD)	60(27)	70(19)	0.07
60 day (n = 49), mean(SD)	71(21)	69(20)	0.7
90 day (n = 49), mean(SD)	76(19)	74(23)	0.76
EQ5D5L index mean (SD)			
30 day (n = 53)	0.37(0.38)	0.48(0.39)	0.24
60 day (n = 49)	0.48(0.44)	0.61(0.36)	0.29
90 day (n = 49)	0.49(0.45)	0.58(0.44)	0.33

The two treatment groups did not differ in cognitive outcomes assessed with MoCA scores. Average MoCA scores measured at 30, 60, and 90 days after ictus were 11, 12.7, and 14 for the galantamine group and 13.2, 12.7, and 15.8 for the placebo group (Table 5, Supplementary Fig. 2). For both groups, the majority of cognitive improvements occurred in the earlier phase—between discharge and 60 days after ictus—and the magnitude of cognitive improvement was also higher during the earlier stage. MoCA scores improved by 3.9 and 4.42 points between discharge and 30 days after ictus for the galantamine and placebo groups, respectively, whereas the magnitude of improvement was 0.8 and 0.7 at the later stage (Supplementary Figs. 3 and 4). No statistical differences were found in all the analyzed aspects of cognitive outcome.

Patients in the galantamine group expressed significantly greater improvement in their QOL measured with the EQ5D5L VAS scores during the early phase (between day 30 and day 60) in comparison to the placebo group. On average, the EQ5D5L VAS score improved by 10.7 points in the galantamine group, whereas the average score decreased by 1.04 points in the placebo group (*p* value < 0.05) (Supplementary Figs. 5, 6 and 7). Improvement in QOL at the later stages of the disease was higher in the galantamine arm but did not reach significance. Each arm showed 6.1-point and 5.8-point increases in QOL. In the galantamine group, consistently higher proportion of patients achieved improvements in QOL, with 57%, between 30 and 60 days after ictus, and 53%, between 60 and 90 days after ictus, expressing improved QOL, whereas 36% and 52% expressed improved QOL in the placebo group. However, the difference did not reach statistical significance (*p* > 0.05) (Supplementary Figs. 6 and 7). Despite these differences in the temporal changes in QOL between the two intervention arms, the absolute EQ5D5L VAS values and EQ5DL indices did not differ at any time point (Table 5, Supplementary Fig. 5).

Post hoc analysis showed that the galantamine group was associated with statistically significant improvement in EQ5D5L VAS scores even after adjusting for each study participant’s age, sex, ethnicity, and the HH grade (Supplementary Table 1). The galantamine group was associated with 10.48-point (*p* < 0.05; 95% CI 3.07–17.90) higher interval improvement in EQ5D5L VAS scores for a given 30-day period. Interaction analysis did not reveal any interactions between the treatment arms and disease stages (Supplementary Table 1).

### Ancillary Outcomes

Cytokine levels measured in serum and CSF across multiple time points did not show consistent differences between treatment groups (Supplementary Fig. 8). In serum, a few cytokines—such as GM-CSF at T3 (*p* = 0.035), MCP-1 at T1 (*p* = 0.041), MIP-1α at T1 and T5 (*p* = 0.044 and 0.046), and TNF-α at T1 (*p* = 0.044)—showed isolated between-group differences (Supplementary Table 2). However, these were not sustained across other time points. Similarly, in CSF, the placebo arm exhibited elevated levels of MIP-1α (T1, *p* = 0.011; T5, *p* = 0.024), MIP-1β (T3, *p* = 0.020; T5, *p* = 0.032), PDGF-AA (T1, *p* = 0.033), and TNF-α (T5, *p* = 0.046) (Supplementary Table 3). These differences were limited to specific time points and did not reflect a consistent treatment effect.

## Discussion

This is a pilot clinical trial to assess the safety, tolerability and preliminary efficacy of galantamine for the treatment of SAH in the acute and chronic stages. Galantamine was found to be safe and well tolerated. The overall rate of intolerability was similar in both arms and did not show differences in the incidence of adverse events and mortality. The galantamine and placebo group did not show differences in mRS and MoCA scores measured at all disease stages—30, 60, and 90 days after ictus. Although the absolute EQ5D5L VAS scores were not different between groups, study participants in the galantamine group showed greater improvement in EQ5D5L VAS scores over time.

Galantamine is a widely used, competitive and reversible AChEi that can modulate postsynaptic nicotinic receptors. Several studies have reported the incidence of adverse events, represented by gastrointestinal symptoms, to be higher in galantamine than other AChEi such as donepezil [18]. The incidence of observed adverse events is dose-dependent, especially high incidence during the initiation phase, and decreases as the treatment period extends [19]. For nausea and vomiting—the most commonly reported adverse events—the incidence rates are up to 23% within 6 months of starting the treatment and decrease to 6% after 24 months [19]. Galantamine is generally considered a well-tolerated agent, with higher than 85% of tolerability [20]. A relatively high incidence of bradycardia observed in our study should be noted, contrasting the current literature reporting its incidence to be lower than or equal to 3% in patients with Alzheimer disease or vascular dementia, and 6% in patients with mild cognitive impairment, respectively [19, 21]. In SAH, bradycardia is a well-known systemic complication with varying incidences, ranging from 1 to 32% [22]. The incidence of cardiac autonomic dysregulation increases as the severity of SAH increases [23]. This study focused on patients with severe SAH, which might have contributed to the high incidence rate of bradycardia. Furthermore, as bradycardia was seen in similar proportion in the placebo arm—with no statistical difference in its incident rates between two treatment arms (*p* = 0.91) (Table 4)—it is likely a complication from SAH and not from treatment with galantamine [24]. However, as an AChEi, galantamine can cause cholinergic access, lead to bradycardia—albeit low reported incident rates—and increase the risk of hospitalization and medical visits; bradycardia should be closely monitored when administering galantamine in patients with SAH [25].

This is the first study to examine the impact of galantamine on mRS and MoCA in patients with SAH. Survivors with SAH are subject to 2.6 times higher risk of dementia, and more than half of the survivors fail to return to their premorbid social roles [26]. Several studies report that AChEi improves memory functions in patients with traumatic brain injury, but their actual effects have not been rigorously examined [27, 28]. In SAH, only one study examined the efficacy of AChEi targeting cognitive using rivastigmine, but the study was performed in the chronic setting, was underpowered, and lacked randomization [29]. The present study has extensively examined galantamine’s side effect profiles and impact on functional and cognitive outcomes in SAH. In particular, this study has established the between-group differences in various functional outcome measures, including mRS, MoCA, and EQ5D5L VAS and index values, at multiple time points, which could serve as a prior in designing future studies. For planning of future studies if a good functional outcome at 90 days (mRS < 2) is to be used as the efficacy outcome measure—with the observed 20% absolute increase in the favorable outcome in the galantamine group and Cohen’s h of 0.41—we estimate enrolling 255 patients in each treatment arm to achieve 80% statistical power at α of 0.05 to examine the superiority of galantamine over placebo.

Despite a limited statistical power due to its nature as a pilot study, this study suggests that galantamine could offer more improvement in EQ5D5L VAS scores over time compared with a placebo. However, the observed improvement was marginally higher than the minimally important difference of EQ5D5L VAS score reported in the literature. Minimally important difference for EQ5D5L VAS varies considerably depending on the patient populations, with the median of 9.0 (IQR 4.0) for distribution-based approach and 8.2 (IQR 8.0) for anchor-based approach [30]. Therefore, the clinical significance of this improvement in EQ5D5L VAS score warrants validation in larger, adequately powered studies. Lack of efficacy in terms of other outcome measures should be taken into account while considering the inherent limitations of the measures. Minimal detectable changes in MoCA have been reported to be as large as 5 points [31]. Although galantamine has been shown to improve cognitive deficits, the absolute magnitude of the treatment effect of galantamine has never been substantial, with the representative clinical trial showing 2.7-point changes in the ADAS-cog scale, out of 70 total points, at 6 months after treatment [32]. Because this was a pilot study, we were underpowered to detect these differences. Extended clinical trials have reported the cognitive benefit of galantamine to last as long as 2 years after the initiation, with a peak cognitive function observed between 3 to 6 months after the treatment [20, 33]. Yet, SAH and neurodegenerative diseases show substantially different clinical courses, alluding to different drug-disease relationships. The observed clinical benefit of galantamine in terms of QOL might be explained by the fact that the clinical benefits of galantamine have not been confined to cognitive functions—studies report galantamine has resulted in an overall improvement in the activities of daily living, behavioral disturbances, and caregiver stress [34, 35]. Potentiation of multiple neurotransmitters through allosteric modulation of nicotinic receptors and enhanced thalamofrontal projection resulting in improved frontal lobe functions have been hypothesized to underlie these benefits [35]. This global improvement in neurological functions might have served as the basis of differential improvement in EQ5D5L VAS scores. Three secondary outcome measures analyzed in this study reflect different yet correlated aspects of functional status. mRS reflects patients’ functional independence, MoCA assesses cognitive functions, and EQ5D5L VAS reflects patient-perceived QOL. The measures show generally good correlations with each other in patients with stroke, but studies specifically focusing on patients with SAH are lacking [36–38]. For future studies evaluating the efficacy of galantamine in patients with SAH, it is recommended that mRS be used as the primary efficacy measure due to its universal usage and well-known properties in patients with SAH and that MoCA and EQ5D5L VAS be assessed as secondary measures.

In contrary to our expected findings, proinflammatory cytokines did not show differential expression between the treatment arms. Our preclinical study showed galantamine can activate downstream JAK2/STAT3 and IRAK-M pathway, reduce systemic inflammation, and improve behavioral outcomes in mice [5]. Systemic anti-inflammation through stimulating α7 nAChR receptors has also been shown to improve outcomes in preclinical models of other systemic inflammatory diseases like sepsis [39]. These benefits are hypothesized to result from galantamine’s ability to enhance cholinergic stimulation through the vagus nerve [10]. However, studies often report depressed sympathetic tone in patients with SAH [23]. It is considered our body’s effort to tame the autonomic storm and catecholamine toxicity resulting from extravasation of hemolytic products and disruption of the blood–brain barrier [40]. Evidence of enhanced vagal tone or depressed sympathetic tone in SAH is also evident in the present study with unusually high rate of bradycardia. The absence of differences in cytokines might have resulted from the lack of additional benefit from further depressing sympathetic tone when the system is already in a depressed state. Experimental factors must also be considered when interpreting our findings. The point-based cytokine measurements were collected at relatively long intervals—at least 24 h apart—which may have limited the sensitivity in detecting dynamic fluctuations in inflammatory responses. Inflammatory markers are known to vary considerably over short time scales, and the current sampling strategy may have been insufficient to fully capture these rapid changes. Employing continuous or more frequent sampling methods could provide a more comprehensive picture of galantamine’s immunomodulatory effects in future studies. Furthermore, systemic disturbances following SAH may have impacted the pharmacokinetics and pharmacodynamics of orally administered medications. To better account for this, future trials should incorporate concurrent measurement of serum galantamine levels, which would help accurately assess the medication’s effect.

This study has important limitations. First, as the study was originally designed as a pilot study, this study is not powered to draw any conclusions about the clinical efficacy of galantamine. Findings with respect to functional, cognitive, and QOL outcomes can serve as a prior for future studies, but neither superiority nor noninferiority can be concluded. Even for safety profiles, since enrollment was not based on preplanned sample calculation, the current study has low statistical power of 25% for any adverse events associated with the investigational product. Based on the previously reported incidence rates of any adverse events due to galantamine, it would require 508 study participants with 1:1 intervention-to-control ratio to achieve statistical power of 80% at α of 0.05 [41]. Second, this study focused only on patients with relatively severe SAH with the Fisher grade 3. This restrictive inclusion criteria limits the generalizability of our findings. Exclusion of patients with mild SAH might have resulted in underestimating the incidence rate of complications or efficacy of the treatment. In addition, the relationship between galantamine and tolerability, safety, and outcomes from patients with the most severe SAH remains unknown. Third, follow-up periods were relatively short. Studies report the duration of post-SAH functional and cognitive complications to be longer than our current understanding [42]. Cognitive impairment due to SAH is observed to last as long as 2.75 years—limited by the length of observation—and the actual impact might be much longer-lasting [43]. Considering the potential benefits of long-term galantamine in other diseases, longer treatment might have resulted in a different finding. Fourth, the relatively large exclusion of a specific group of patients, or the conduct of an interim analysis may have impacted study generalizability or enrollment dynamics. However, it should be noted that, throughout this study, all patients were prospectively enrolled following a strictly uniform protocol, and the trial procedures remained identical before and after the interim analysis.

## Conclusions

Subarachnoid hemorrhage causes life-long morbidity, including functional and cognitive impairment leading to poor QOL. Galantamine has potential to ameliorate the damage. This multicenter, double-masked, randomized, placebo-controlled pilot clinical trial investigated tolerability and safety of galantamine in SAH. Galantamine was well tolerable and safe to administer in patients with SAH during early and late stages of the disease. Further studies are necessary to validate the tolerability and safety of galantamine and investigate its efficacy in SAH.

## Supplementary Information

Below is the link to the electronic supplementary material.

Supplementary file1 (PNG 683 KB)

Supplementary file2 (PNG 153 KB)

Supplementary file3 (PNG 210 KB)

Supplementary file4 (PNG 1593 KB)

Supplementary file5 (PNG 143 KB)

Supplementary file6 (PNG 154 KB)

Supplementary file7 (PNG 1449 KB)

Supplementary file8 (PNG 676 KB)

Supplementary file9 (DOCX 58 KB)
