# Supplementary figures and images for: SAHRANG: Subarachnoid Hemorrhage Recovery and Galantamine: A Pilot Multicenter Randomized Placebo-Controlled Trial
Source: Neurocrit Care. 2025 Aug 28;43(3):986–98. doi: 10.1007/s12028-025-02349-3 (PMC12647330; doi:10.1007/s12028-025-02349-3)

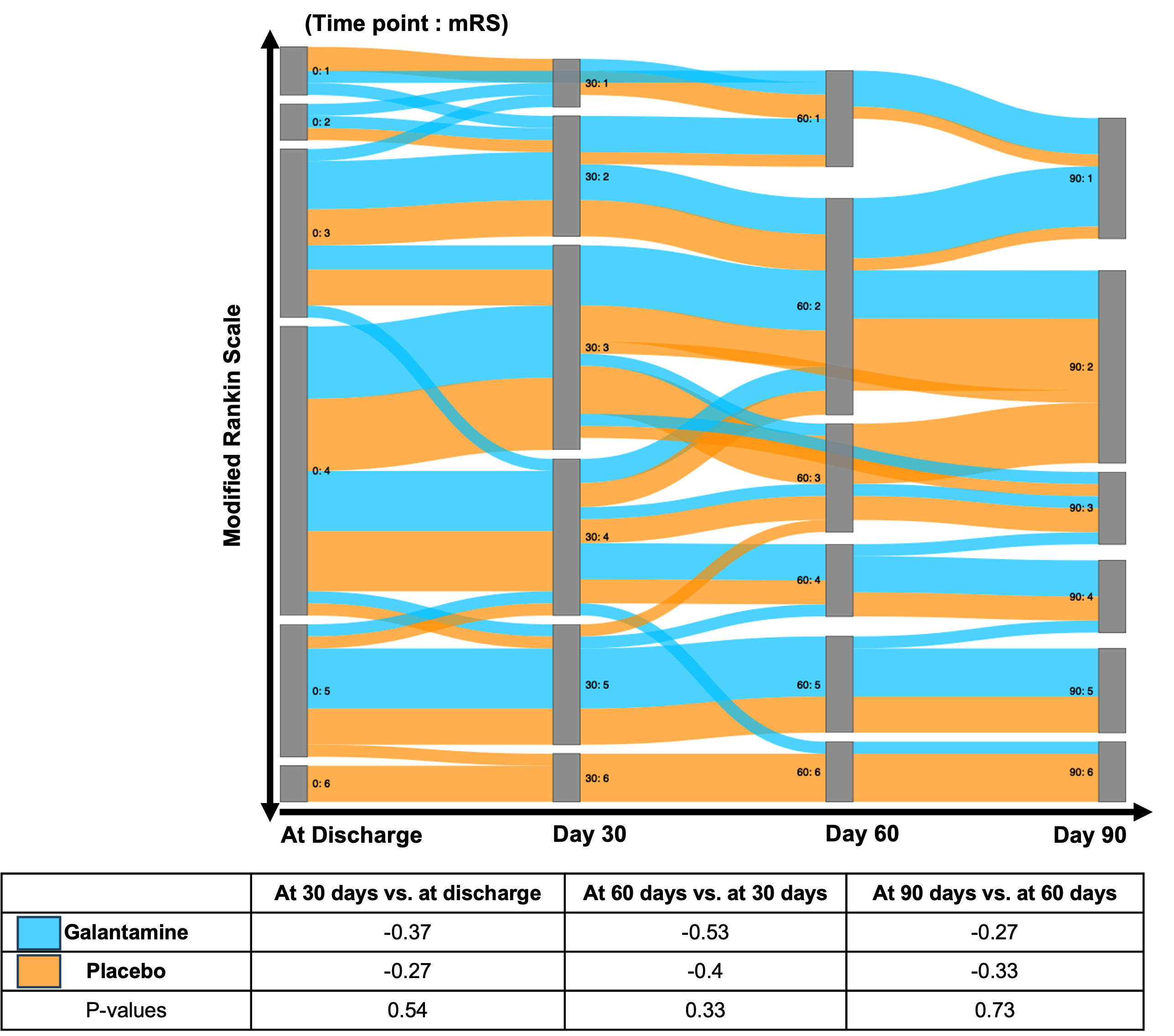

Supplement: Supplementary file 1 — Supplementary file1 (PNG 683 KB) [file 12028_2025_2349_MOESM1_ESM.png]

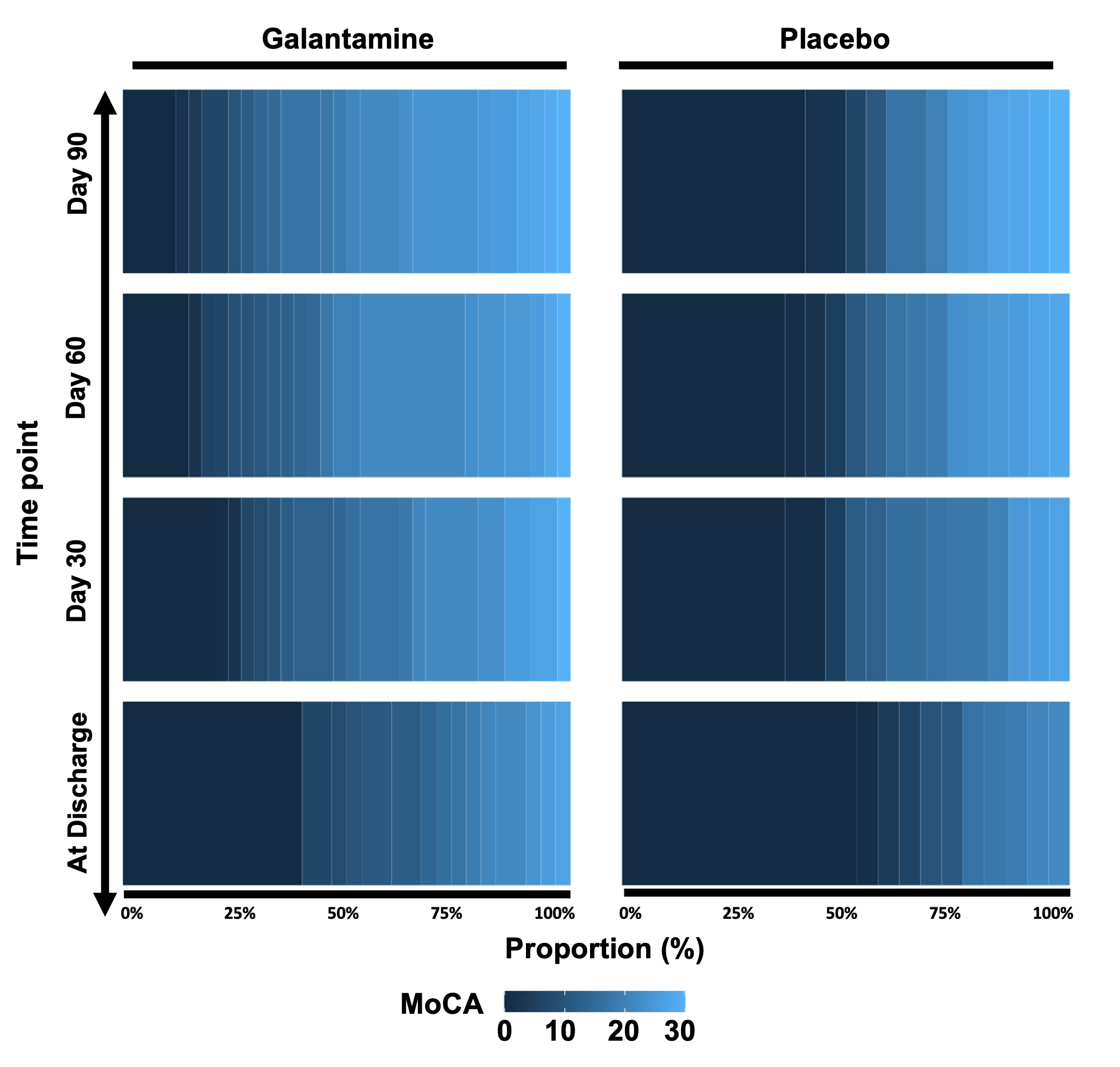

Supplement: Supplementary file 2 — Supplementary file2 (PNG 153 KB) [file 12028_2025_2349_MOESM2_ESM.png]

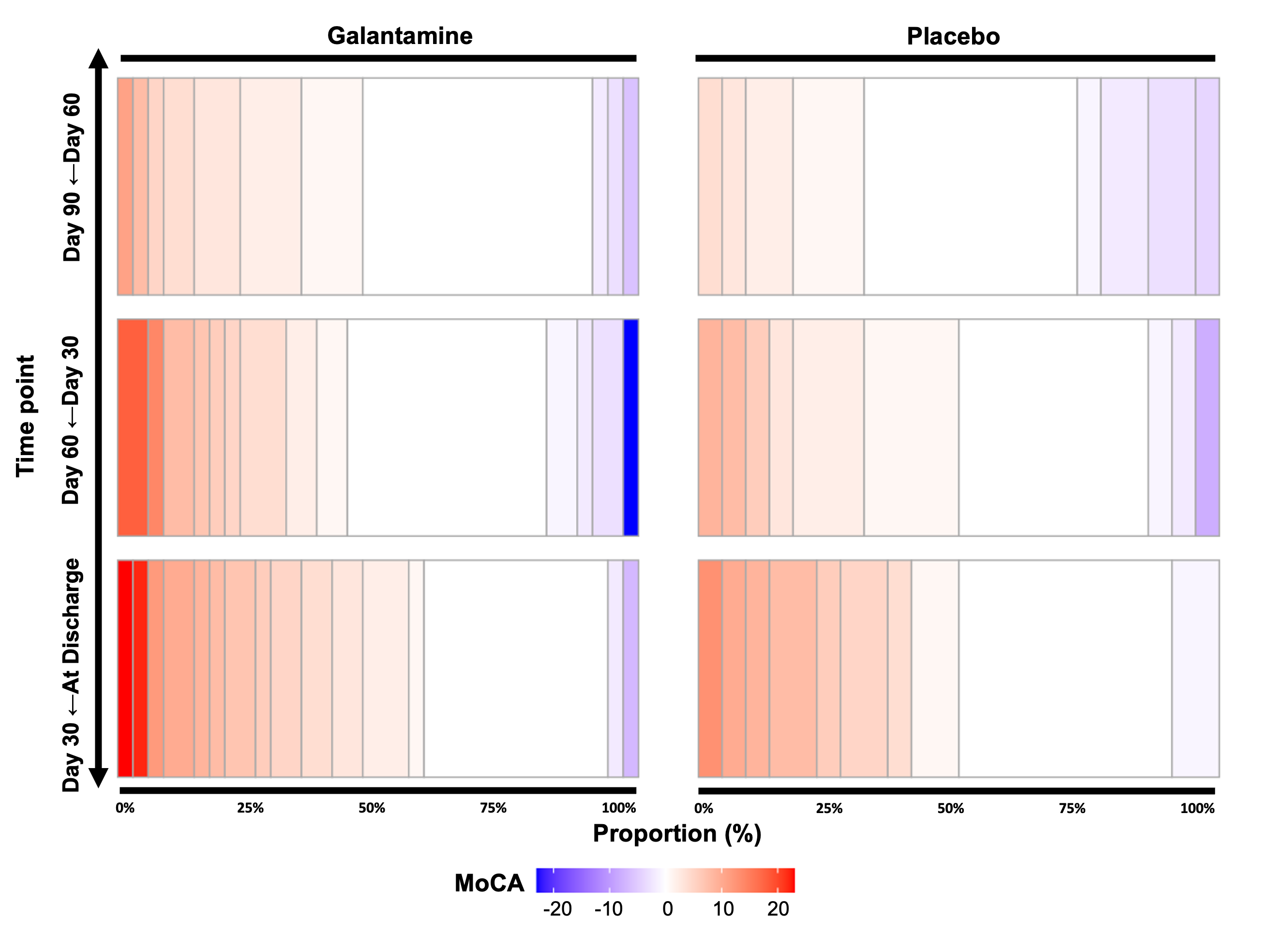

Supplement: Supplementary file 3 — Supplementary file3 (PNG 210 KB) [file 12028_2025_2349_MOESM3_ESM.png]

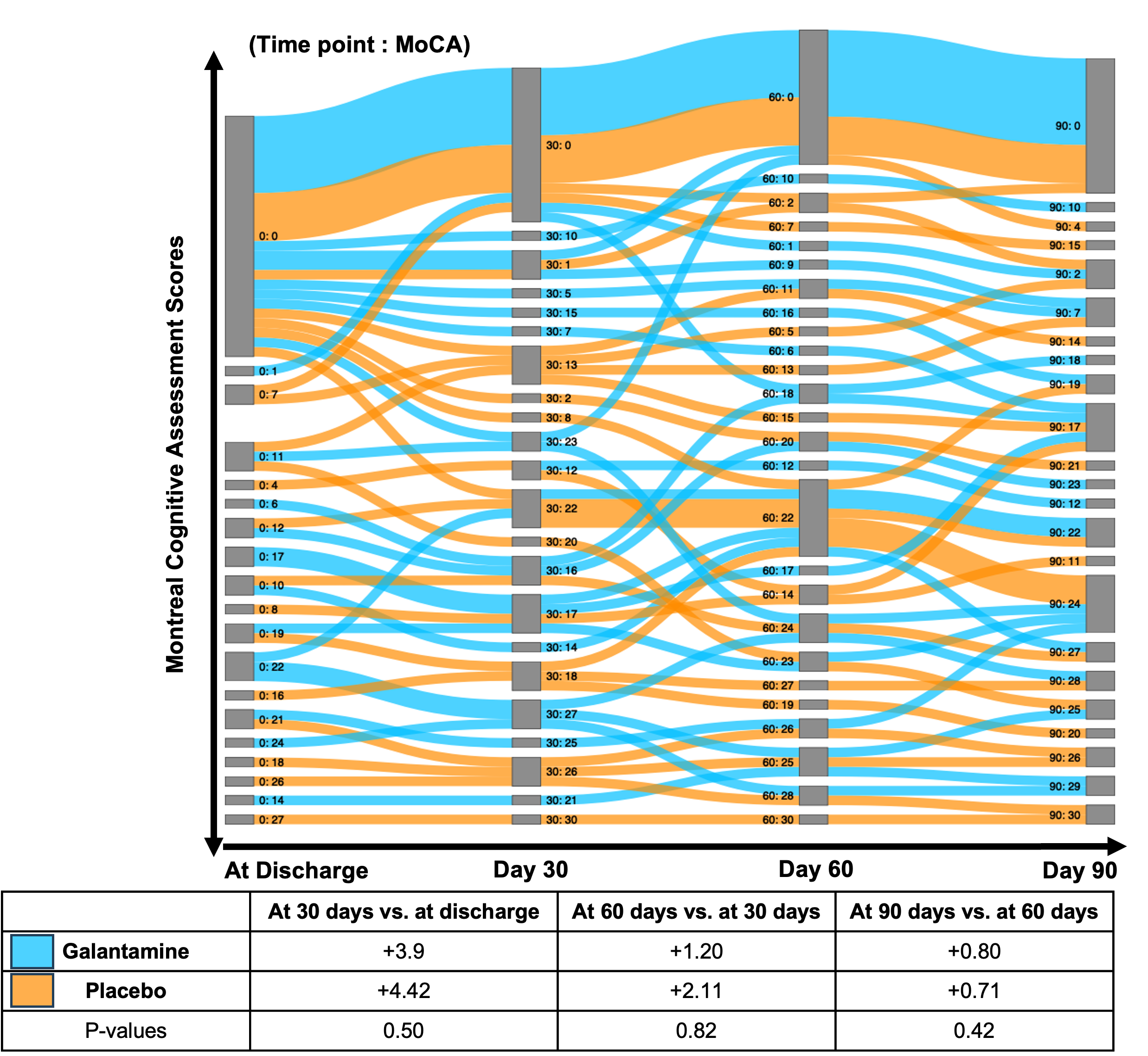

Supplement: Supplementary file 4 — Supplementary file4 (PNG 1593 KB) [file 12028_2025_2349_MOESM4_ESM.png]

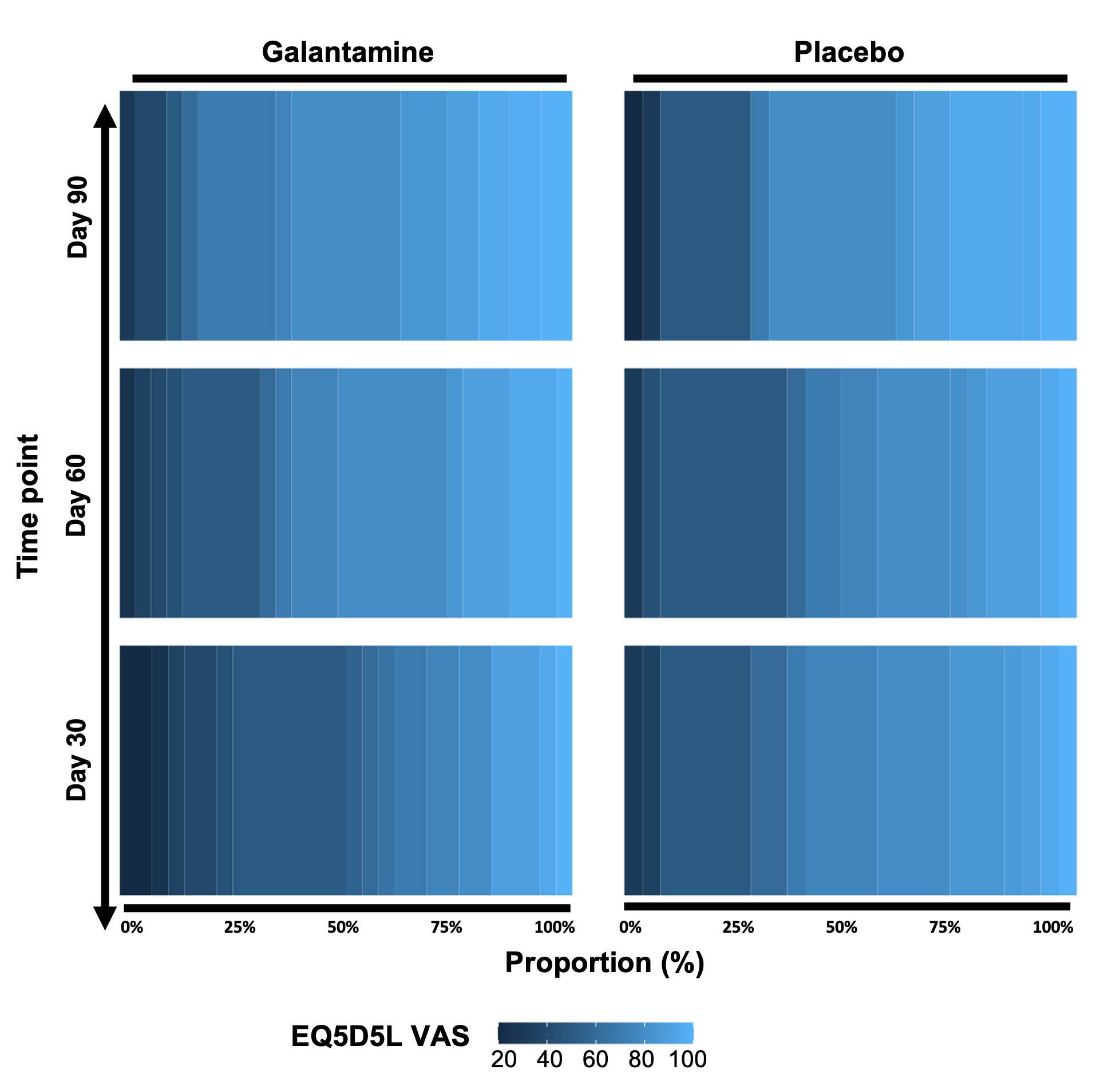

Supplement: Supplementary file 5 — Supplementary file5 (PNG 143 KB) [file 12028_2025_2349_MOESM5_ESM.png]

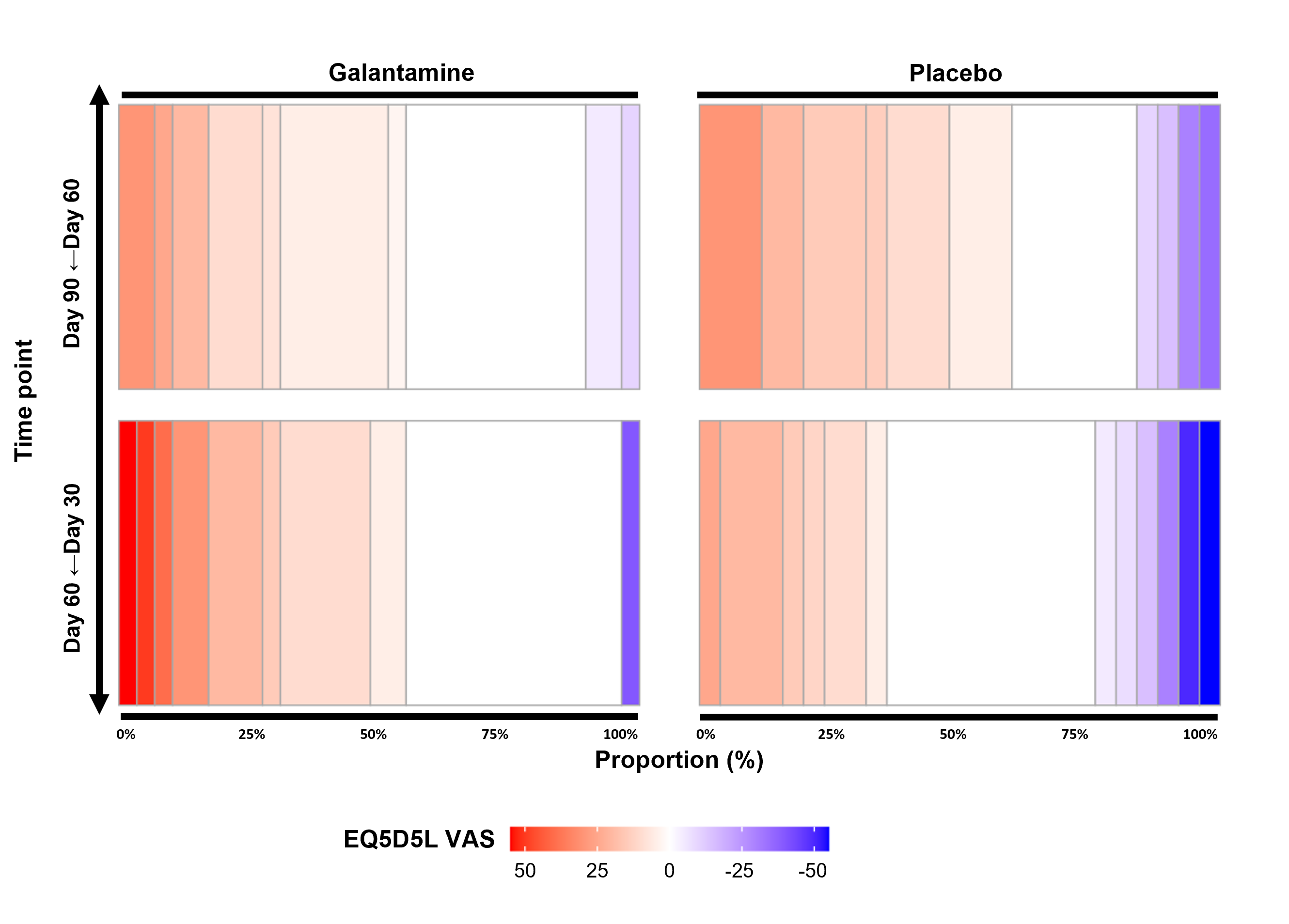

Supplement: Supplementary file 6 — Supplementary file6 (PNG 154 KB) [file 12028_2025_2349_MOESM6_ESM.png]

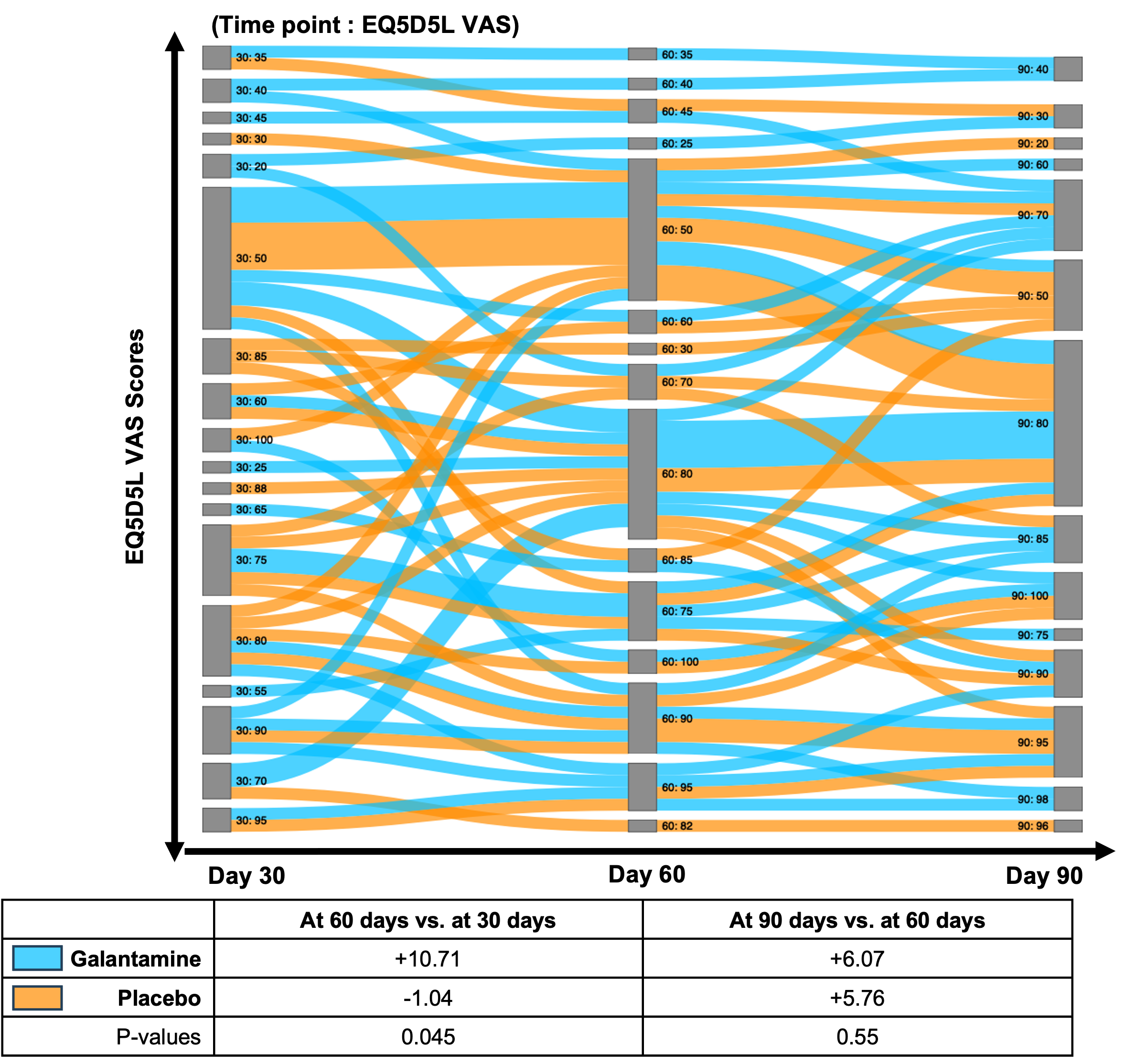

Supplement: Supplementary file 7 — Supplementary file7 (PNG 1449 KB) [file 12028_2025_2349_MOESM7_ESM.png]

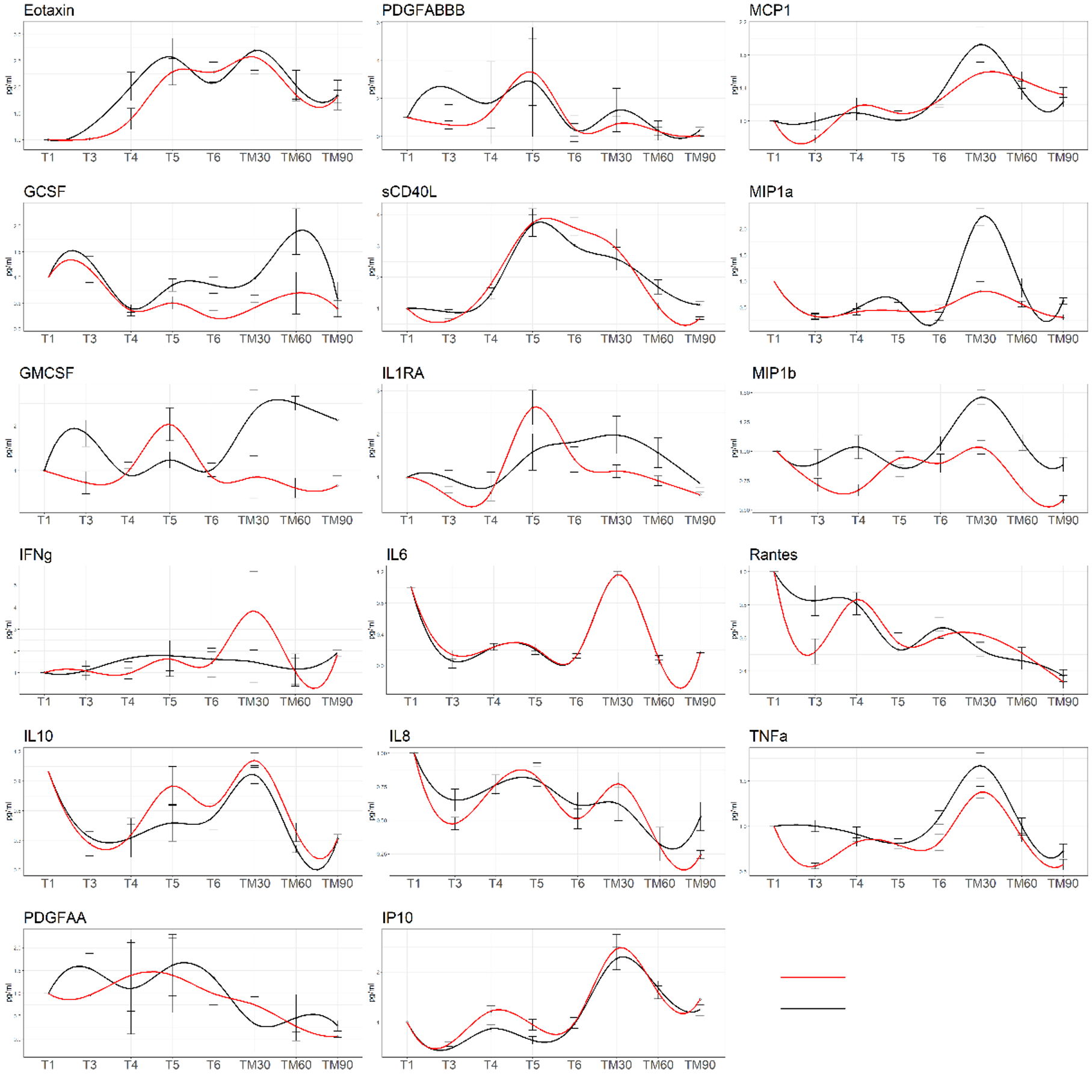

Supplement: Supplementary file 8 — Supplementary file8 (PNG 676 KB) [file 12028_2025_2349_MOESM8_ESM.png]
