# Supplementary material for: SAHRANG: Subarachnoid Hemorrhage Recovery and Galantamine: A Pilot Multicenter Randomized Placebo-Controlled Trial
Source: Neurocrit Care. 2025 Aug 28;43(3):986–98. doi: 10.1007/s12028-025-02349-3 (PMC12647330; doi:10.1007/s12028-025-02349-3)
Supplement: Supplementary file 9 — Supplementary file9 (DOCX 58 KB) [file 12028_2025_2349_MOESM9_ESM.docx]

**Supplementary Table 1. Effects of treatment groups and disease stages on interval changes in EQ5D5L VAS**

|  | | **Model 1** (without interaction) | | | **Model 2** (with interaction) | | |
| --- | --- | --- | --- | --- | --- | --- | --- |
|  | | **Estimate** | **95% CI** | **p-val** | **Estimate** | **95% CI** | **p-val** |
| **Patient factors** | |  | | |  | | |
| Age | | 0.32 | 0.02 – 0.62 | 0.04 | 0.31 | 0.02 – 0.60 | 0.04 |
| Sex (Female) | | 3.68 | -3.97 – 11.34 | 0.35 | 3.77 | -3.59 – 11.13 | 0.34 |
| Ethnicity (Non-Hispanic) | | 4.98 | -2.25 – 12.21 | 0.18 | 4.95 | -1.99 – 11.90 | 0.18 |
| Hunt-Hess grade | | -1.87 | -5.69 – 1.95 | 0.34 | -1.96 | -5.63 – 1.72 | 0.32 |
| **Treatment and disease stages** | |  | | |  | | |
| Treatment (Galantamine) | | 10.48 | 3.07 – 17.90 | 0.01 |  |  |  |
| Phase (Early) | | -0.95 | -7.92 – 6.02 | 0.79 |  |  |  |
| **Treatment** * **disease stages** | |  | | |  | | |
| Placebo | Late  (60-90 days) |  |  |  | *Ref*. |  |  |
| Placebo | Early  (30-60 days) |  |  |  | -5.85 | -15.59 – 3.89 | 0.26 |
| Galantamine | Late |  |  |  | 5.63 | -4.36 – 15.62 | 0.29 |
| Galantamine | Early |  |  |  | 9.30 | -4.12 – 22.73 | 0.19 |
|  |  |  |  |  |  |  |  |

**Supplementary Table 2. Serum cytokine levels at different timepoints**

| Cytokine Summary (Serum) | | | | | | |
| --- | --- | --- | --- | --- | --- | --- |
| Median (IQR) and Wilcoxon p-values between Treatment Groups | | | | | | |
| cytokine | Timepoint* | Galantamine (pg/ml) | n | Placebo (pg/ml) | n | p-value |
| eotaxin | t1 | 34.99 (IQR: 34.58) | 29 | 37.98 (IQR: 33.12) | 30 | 0.282 |
| eotaxin | t3 | 38.36 (IQR: 39.17) | 30 | 45.97 (IQR: 39.27) | 30 | 0.820 |
| eotaxin | t4 | 68.98 (IQR: 50.05) | 28 | 64.37 (IQR: 54.34) | 29 | 0.710 |
| eotaxin | t5 | 65.99 (IQR: 60.07) | 29 | 87.3 (IQR: 40.71) | 23 | 0.362 |
| eotaxin | t6 | 79.73 (IQR: 68.87) | 28 | 76.17 (IQR: 41.69) | 22 | 0.977 |
| eotaxin | tm30 | 71.46 (IQR: 55.56) | 23 | 87.34 (IQR: 36.42) | 23 | 0.528 |
| eotaxin | tm60 | 49.69 (IQR: 52.98) | 20 | 62.88 (IQR: 20.69) | 18 | 0.511 |
| eotaxin | tm90 | 52.56 (IQR: 50.57) | 19 | 57.41 (IQR: 26.83) | 17 | 0.949 |
| gcsf | t1 | 49.57 (IQR: 102.04) | 29 | 64.72 (IQR: 94.89) | 30 | 0.835 |
| gcsf | t3 | 52.87 (IQR: 29.9) | 30 | 65.29 (IQR: 40.46) | 29 | 0.596 |
| gcsf | t4 | 16.93 (IQR: 23.37) | 23 | 27.38 (IQR: 40.7) | 25 | 0.992 |
| gcsf | t5 | 42.5 (IQR: 19.27) | 14 | 32.82 (IQR: 26.99) | 13 | 0.068 |
| gcsf | t6 | 75.06 (IQR: 109.78) | 10 | 42.96 (IQR: 24.33) | 7 | 0.157 |
| gcsf | tm30 | 94.08 (IQR: 99.83) | 8 | 71.23 (IQR: 11.34) | 5 | 0.379 |
| gcsf | tm60 | 220.64 (IQR: 364.82) | 7 | 117.07 (IQR: 24.86) | 3 | 0.833 |
| gcsf | tm90 | 40.36 (IQR: 155.61) | 7 | 59.98 (IQR: 13.73) | 4 | 0.788 |
| gmcsf | t1 | 1.29 (IQR: 6.78) | 29 | 2.39 (IQR: 6.71) | 30 | 0.627 |
| gmcsf | t3 | 4.76 (IQR: 4.42) | 23 | 2.69 (IQR: 3.13) | 26 | 0.035 |
| gmcsf | t4 | 2.42 (IQR: 6.65) | 20 | 2.79 (IQR: 4.69) | 27 | 0.796 |
| gmcsf | t5 | 4.2 (IQR: 7.06) | 22 | 4.92 (IQR: 5.68) | 21 | 0.932 |
| gmcsf | t6 | 3.94 (IQR: 8.11) | 19 | 5.34 (IQR: 7.09) | 18 | 0.523 |
| gmcsf | tm30 | 9.89 (IQR: 20.74) | 8 | 6.15 (IQR: 8.88) | 12 | 0.295 |
| gmcsf | tm60 | 16.6 (IQR: 83.66) | 8 | 3.16 (IQR: 1.31) | 6 | 0.651 |
| gmcsf | tm90 | 7.7 (IQR: 44.18) | 7 | 2.63 (IQR: 3.06) | 8 | 0.072 |
| ifng | t1 | 0.74 (IQR: 6.34) | 29 | 0.74 (IQR: 7.16) | 30 | 0.774 |
| ifng | t3 | 3.39 (IQR: 6.04) | 25 | 2.12 (IQR: 4.41) | 25 | 0.404 |
| ifng | t4 | 2.28 (IQR: 5.97) | 24 | 2.03 (IQR: 3.01) | 28 | 0.532 |
| ifng | t5 | 3.13 (IQR: 4.96) | 25 | 6.18 (IQR: 6.61) | 19 | 0.319 |
| ifng | t6 | 2.13 (IQR: 6.58) | 24 | 4.71 (IQR: 7.71) | 19 | 0.104 |
| ifng | tm30 | 4.21 (IQR: 8.16) | 19 | 6.24 (IQR: 13.89) | 21 | 0.424 |
| ifng | tm60 | 4.54 (IQR: 95.97) | 16 | 3.3 (IQR: 4.85) | 14 | 0.478 |
| ifng | tm90 | 8.1 (IQR: 32.35) | 14 | 4.88 (IQR: 7.67) | 11 | 0.410 |
| il10 | t1 | 11.47 (IQR: 47.06) | 29 | 4.73 (IQR: 40.37) | 30 | 0.943 |
| il10 | t3 | 5.01 (IQR: 10.55) | 27 | 6.57 (IQR: 9.15) | 21 | 0.540 |
| il10 | t4 | 8.32 (IQR: 9.31) | 20 | 14.15 (IQR: 21.21) | 18 | 0.203 |
| il10 | t5 | 6.81 (IQR: 7.07) | 26 | 8.35 (IQR: 11.7) | 23 | 0.865 |
| il10 | t6 | 7.04 (IQR: 6.12) | 21 | 8.35 (IQR: 11.82) | 15 | 0.089 |
| il10 | tm30 | 13.85 (IQR: 10.55) | 10 | 20.06 (IQR: 10.02) | 11 | 0.526 |
| il10 | tm60 | 4.45 (IQR: 9.77) | 16 | 2.49 (IQR: 5.53) | 12 | 0.377 |
| il10 | tm90 | 5.24 (IQR: 3.93) | 11 | 10.97 (IQR: 23.47) | 5 | 0.364 |
| il1ra | t1 | 25.62 (IQR: 47.47) | 29 | 35.3 (IQR: 41.91) | 30 | 0.386 |
| il1ra | t3 | 23.62 (IQR: 39.99) | 30 | 17.67 (IQR: 10.24) | 30 | 0.304 |
| il1ra | t4 | 30.32 (IQR: 55.72) | 28 | 17.05 (IQR: 35.11) | 30 | 0.732 |
| il1ra | t5 | 47.77 (IQR: 44.55) | 29 | 64.67 (IQR: 55.54) | 23 | 0.194 |
| il1ra | t6 | 44.51 (IQR: 38.71) | 28 | 51.67 (IQR: 50.18) | 22 | 0.822 |
| il1ra | tm30 | 53.68 (IQR: 76.96) | 21 | 37.22 (IQR: 46.73) | 22 | 0.688 |
| il1ra | tm60 | 34.35 (IQR: 40.98) | 20 | 25.87 (IQR: 8.12) | 17 | 0.493 |
| il1ra | tm90 | 23.35 (IQR: 26.56) | 19 | 16.56 (IQR: 9.89) | 17 | 0.874 |
| il6 | t1 | 5.88 (IQR: 0) | 29 | 5.88 (IQR: 70.64) | 30 | 0.157 |
| il6 | t3 | 28.86 (IQR: 27.22) | 14 | 43.8 (IQR: 65.43) | 16 | 0.423 |
| il6 | t4 | 18.81 (IQR: 46.3) | 13 | 34.79 (IQR: 74.2) | 15 | 0.447 |
| il6 | t5 | 10.17 (IQR: 13.57) | 18 | 15.87 (IQR: 20.87) | 15 | 0.240 |
| il6 | t6 | 18.26 (IQR: 15.6) | 13 | 11.43 (IQR: 22.97) | 12 | 0.769 |
| il6 | tm30 | 35.12 (IQR: 53.16) | 7 | 79.05 (IQR: 58.83) | 6 | 0.534 |
| il6 | tm60 | 12.83 (IQR: 50.96) | 10 | 17.98 (IQR: 64.21) | 5 | 0.854 |
| il6 | tm90 | 37.17 (IQR: 72.52) | 7 | 4.19 (IQR: 12.2) | 6 | 0.138 |
| il8 | t1 | 7.67 (IQR: 8.29) | 29 | 9.96 (IQR: 16.64) | 30 | 0.367 |
| il8 | t3 | 4.63 (IQR: 8.73) | 28 | 9.46 (IQR: 10.41) | 26 | 0.092 |
| il8 | t4 | 6.62 (IQR: 6.79) | 28 | 8.92 (IQR: 12.61) | 29 | 0.285 |
| il8 | t5 | 8.44 (IQR: 9.44) | 27 | 9.64 (IQR: 13.72) | 23 | 0.475 |
| il8 | t6 | 7.18 (IQR: 7.05) | 23 | 7.18 (IQR: 7.62) | 22 | 0.454 |
| il8 | tm30 | 13.29 (IQR: 13.24) | 13 | 9.82 (IQR: 14.9) | 18 | 0.307 |
| il8 | tm60 | 5.85 (IQR: 18.83) | 16 | 3.11 (IQR: 6.14) | 15 | 0.353 |
| il8 | tm90 | 3.83 (IQR: 13.16) | 16 | 3.89 (IQR: 2.96) | 13 | 0.812 |
| ip10 | t1 | 377.89 (IQR: 393.01) | 29 | 287.9 (IQR: 271.5) | 30 | 0.328 |
| ip10 | t3 | 169.1 (IQR: 188.38) | 30 | 166.16 (IQR: 115.52) | 30 | 0.878 |
| ip10 | t4 | 242.16 (IQR: 307.97) | 28 | 299.45 (IQR: 333.75) | 30 | 0.605 |
| ip10 | t5 | 203.04 (IQR: 177.3) | 29 | 299.45 (IQR: 326.74) | 23 | 0.257 |
| ip10 | t6 | 300.33 (IQR: 290.86) | 28 | 288.2 (IQR: 128.56) | 22 | 0.564 |
| ip10 | tm30 | 707.38 (IQR: 856.91) | 23 | 569.35 (IQR: 510.91) | 23 | 0.407 |
| ip10 | tm60 | 458.21 (IQR: 312.14) | 20 | 380.87 (IQR: 266.41) | 18 | 0.553 |
| ip10 | tm90 | 461.4 (IQR: 183.11) | 19 | 422.57 (IQR: 302.61) | 17 | 0.510 |
| mcp1 | t1 | 153.81 (IQR: 98.7) | 29 | 182.8 (IQR: 206.38) | 30 | 0.041 |
| mcp1 | t3 | 150.48 (IQR: 69.74) | 30 | 128.67 (IQR: 115.88) | 30 | 0.912 |
| mcp1 | t4 | 184.07 (IQR: 130.43) | 28 | 194.32 (IQR: 188.35) | 30 | 0.374 |
| mcp1 | t5 | 175.67 (IQR: 152.11) | 29 | 212.83 (IQR: 120.35) | 23 | 0.114 |
| mcp1 | t6 | 200.91 (IQR: 152.55) | 28 | 213.54 (IQR: 109.88) | 22 | 0.900 |
| mcp1 | tm30 | 276.83 (IQR: 135.16) | 23 | 276.48 (IQR: 209.32) | 23 | 1.000 |
| mcp1 | tm60 | 204.97 (IQR: 56.44) | 20 | 220.58 (IQR: 57.98) | 18 | 0.478 |
| mcp1 | tm90 | 171.69 (IQR: 30.39) | 19 | 174.73 (IQR: 63.24) | 17 | 1.000 |
| mip1a | t1 | 0.39 (IQR: 4.16) | 29 | 4.3 (IQR: 6.51) | 30 | 0.044 |
| mip1a | t3 | 1.21 (IQR: 3.77) | 15 | 2.9 (IQR: 5.8) | 15 | 0.852 |
| mip1a | t4 | 3.27 (IQR: 5.1) | 17 | 1.48 (IQR: 4.88) | 22 | 0.955 |
| mip1a | t5 | 0.67 (IQR: 1.92) | 27 | 1.45 (IQR: 1.44) | 23 | 0.046 |
| mip1a | t6 | 1.67 (IQR: 2.87) | 15 | 1.18 (IQR: 3.16) | 20 | 0.867 |
| mip1a | tm30 | 4.46 (IQR: 3.93) | 10 | 4.33 (IQR: 1.63) | 15 | 0.956 |
| mip1a | tm60 | 2.79 (IQR: 9.93) | 13 | 1.17 (IQR: 2.59) | 14 | 0.103 |
| mip1a | tm90 | 4.2 (IQR: 4.93) | 11 | 0.82 (IQR: 1.8) | 9 | 0.182 |
| mip1b | t1 | 24.39 (IQR: 15.07) | 29 | 36.66 (IQR: 42.39) | 30 | 0.060 |
| mip1b | t3 | 30.2 (IQR: 19.43) | 30 | 21.49 (IQR: 18.43) | 30 | 0.432 |
| mip1b | t4 | 25.94 (IQR: 18.63) | 28 | 24.33 (IQR: 18.18) | 30 | 0.311 |
| mip1b | t5 | 24.9 (IQR: 20.42) | 29 | 31.46 (IQR: 15.84) | 23 | 0.428 |
| mip1b | t6 | 29.18 (IQR: 13.25) | 28 | 27.46 (IQR: 10.92) | 22 | 0.792 |
| mip1b | tm30 | 31.88 (IQR: 14.02) | 23 | 31.8 (IQR: 19.33) | 23 | 0.939 |
| mip1b | tm60 | 24.7 (IQR: 16.72) | 20 | 18.43 (IQR: 11.34) | 18 | 0.254 |
| mip1b | tm90 | 20.55 (IQR: 13.62) | 19 | 18.46 (IQR: 7.29) | 17 | 0.594 |
| pdgfaa | t1 | 82.91 (IQR: 238.02) | 29 | 144.2 (IQR: 297.44) | 30 | 0.500 |
| pdgfaa | t3 | 190.75 (IQR: 356.56) | 30 | 76.89 (IQR: 225.43) | 28 | 0.275 |
| pdgfaa | t4 | 152.68 (IQR: 532.24) | 28 | 96.27 (IQR: 446.58) | 29 | 0.943 |
| pdgfaa | t5 | 156.76 (IQR: 626.96) | 29 | 228.43 (IQR: 759.17) | 23 | 0.648 |
| pdgfaa | t6 | 153.2 (IQR: 587.63) | 28 | 169.03 (IQR: 689.34) | 22 | 1.000 |
| pdgfaa | tm30 | 89.16 (IQR: 435.77) | 18 | 103.57 (IQR: 429.87) | 18 | 0.563 |
| pdgfaa | tm60 | 62.96 (IQR: 95.53) | 18 | 49.96 (IQR: 128.49) | 14 | 0.837 |
| pdgfaa | tm90 | 31.95 (IQR: 88.82) | 17 | 26.46 (IQR: 88.32) | 12 | 0.811 |
| pdgfabbb | t1 | 515.57 (IQR: 1565.6) | 29 | 920.44 (IQR: 1830.34) | 30 | 0.417 |
| pdgfabbb | t3 | 1884.53 (IQR: 4151.9) | 28 | 809.25 (IQR: 1280.78) | 22 | 0.232 |
| pdgfabbb | t4 | 1455.85 (IQR: 11147.25) | 27 | 825 (IQR: 5553.67) | 30 | 0.305 |
| pdgfabbb | t5 | 2210.77 (IQR: 10119.87) | 29 | 3925.22 (IQR: 11560.3) | 22 | 0.699 |
| pdgfabbb | t6 | 1651.36 (IQR: 13597.94) | 15 | 5263.01 (IQR: 10561.49) | 13 | 0.717 |
| pdgfabbb | tm30 | 21161.95 (IQR: 12342.02) | 3 | 18929.01 (IQR: 1858.78) | 2 | 0.800 |
| pdgfabbb | tm60 | 507.12 (IQR: 1930.45) | 11 | 1398.11 (IQR: 1473.35) | 10 | 0.918 |
| pdgfabbb | tm90 | 916.38 (IQR: 2127.12) | 11 | 544.45 (IQR: 1441.58) | 7 | 0.856 |
| rantes | t1 | 962.71 (IQR: 723.66) | 29 | 1142.25 (IQR: 640.71) | 30 | 0.316 |
| rantes | t3 | 833.8 (IQR: 534.51) | 30 | 644.4 (IQR: 749.47) | 29 | 0.354 |
| rantes | t4 | 837.14 (IQR: 699.15) | 28 | 1163.26 (IQR: 735.27) | 30 | 0.671 |
| rantes | t5 | 671.45 (IQR: 440.49) | 29 | 663.52 (IQR: 370.05) | 23 | 0.390 |
| rantes | t6 | 721.24 (IQR: 414.42) | 28 | 710.68 (IQR: 559.39) | 22 | 0.915 |
| rantes | tm30 | 554.27 (IQR: 306.59) | 23 | 709.4 (IQR: 298.12) | 23 | 0.229 |
| rantes | tm60 | 511.57 (IQR: 255.69) | 20 | 656.02 (IQR: 784.46) | 18 | 0.654 |
| rantes | tm90 | 440.07 (IQR: 406.39) | 18 | 257.38 (IQR: 429.58) | 16 | 0.281 |
| scd40l | t1 | 95.99 (IQR: 161.4) | 29 | 133.12 (IQR: 87.92) | 30 | 0.820 |
| scd40l | t3 | 85.51 (IQR: 134.63) | 30 | 67.76 (IQR: 52.17) | 30 | 0.072 |
| scd40l | t4 | 195.32 (IQR: 212.28) | 28 | 154.07 (IQR: 295.11) | 30 | 0.726 |
| scd40l | t5 | 410.99 (IQR: 404.19) | 29 | 305.27 (IQR: 283.15) | 23 | 0.847 |
| scd40l | t6 | 318.51 (IQR: 413.58) | 28 | 430.88 (IQR: 574.43) | 22 | 0.747 |
| scd40l | tm30 | 306.81 (IQR: 344.5) | 23 | 428.52 (IQR: 416.25) | 23 | 0.339 |
| scd40l | tm60 | 166.9 (IQR: 196.25) | 20 | 116.01 (IQR: 103.23) | 18 | 0.465 |
| scd40l | tm90 | 118.53 (IQR: 165.32) | 19 | 63.72 (IQR: 52.65) | 17 | 0.310 |
| tnfa | t1 | 10.91 (IQR: 6.42) | 29 | 14.44 (IQR: 9.38) | 30 | 0.044 |
| tnfa | t3 | 9.84 (IQR: 6.13) | 30 | 7.98 (IQR: 5.5) | 30 | 0.626 |
| tnfa | t4 | 10.41 (IQR: 5.64) | 28 | 10.04 (IQR: 7.5) | 30 | 0.779 |
| tnfa | t5 | 10.5 (IQR: 7.56) | 29 | 9.42 (IQR: 8.93) | 23 | 0.306 |
| tnfa | t6 | 11.5 (IQR: 6.83) | 28 | 12.04 (IQR: 5.44) | 22 | 0.961 |
| tnfa | tm30 | 16.58 (IQR: 12.86) | 23 | 17.37 (IQR: 11.01) | 23 | 0.244 |
| tnfa | tm60 | 12.63 (IQR: 9.33) | 20 | 11.31 (IQR: 6.98) | 18 | 0.236 |
| tnfa | tm90 | 8.88 (IQR: 4.53) | 19 | 9.69 (IQR: 5.68) | 17 | 0.899 |

* within 24 hours of admission (T1), 24–48 hours (T3), days 3–5 (T4), days 6–8 (T5), over 8 days but prior to discharge (T6), and at 30 (TM30), 60 (TM60), and 90 (TM90) days post-ictus

**Supplementary Table 3. CSF cytokine levels at different timepoints**

| Cytokine Summary (CSF Only) | | | | | | |
| --- | --- | --- | --- | --- | --- | --- |
| Median (IQR) and Wilcoxon p-values between Treatment Groups | | | | | | |
| cytokine | Timepoint* | Galantamine (pg/ml) | n | Placebo (pg/ml) | n | p-value |
| eotaxin | t1 | 3 (IQR: 6.21) | 18 | 4.27 (IQR: 5.01) | 15 | 0.562 |
| eotaxin | t3 | 2.42 (IQR: 2.65) | 24 | 1.87 (IQR: 2.24) | 18 | 0.868 |
| eotaxin | t4 | 5.24 (IQR: 4.66) | 23 | 6.35 (IQR: 3.45) | 24 | 0.915 |
| eotaxin | t5 | 5.96 (IQR: 2.74) | 23 | 5.44 (IQR: 5.72) | 19 | 0.685 |
| eotaxin | t6 | 7.81 (IQR: 7.69) | 13 | 7.22 (IQR: 1.67) | 6 | 0.538 |
| gcsf | t1 | 39.27 (IQR: 36.57) | 18 | 45.84 (IQR: 81.49) | 15 | 0.303 |
| gcsf | t3 | 27.07 (IQR: 26.02) | 24 | 28.47 (IQR: 40.66) | 18 | 0.819 |
| gcsf | t4 | 21.57 (IQR: 30.95) | 23 | 14.46 (IQR: 28.79) | 24 | 0.221 |
| gcsf | t5 | 46.51 (IQR: 132.54) | 23 | 35.67 (IQR: 152.98) | 19 | 0.817 |
| gcsf | t6 | 8.64 (IQR: 90.51) | 13 | 8.64 (IQR: 6.48) | 6 | 0.761 |
| gmcsf | t1 | 0.23 (IQR: 0) | 18 | 0.23 (IQR: 0) | 15 | 0.638 |
| gmcsf | t3 | 0.47 (IQR: 0.35) | 24 | 0.23 (IQR: 0) | 18 | 0.099 |
| gmcsf | t4 | 0.38 (IQR: 0.73) | 23 | 0.38 (IQR: 0.38) | 24 | 0.911 |
| gmcsf | t5 | 1.19 (IQR: 1.08) | 23 | 1.58 (IQR: 1.86) | 19 | 0.681 |
| gmcsf | t6 | 0.53 (IQR: 0.66) | 13 | 1 (IQR: 0.94) | 6 | 0.591 |
| ifng | t1 | 0.53 (IQR: 0) | 18 | 0.53 (IQR: 0) | 15 | 0.205 |
| ifng | t3 | 0.04 (IQR: 0) | 24 | 0.04 (IQR: 0) | 18 | 0.144 |
| ifng | t4 | 0.04 (IQR: 0.54) | 23 | 0.23 (IQR: 0.86) | 24 | 0.449 |
| ifng | t5 | 0.32 (IQR: 0) | 23 | 0.32 (IQR: 0.3) | 19 | 0.197 |
| ifng | t6 | 0.36 (IQR: 0) | 13 | 0.36 (IQR: 0) | 6 | 1.000 |
| il10 | t1 | 16.9 (IQR: 36.65) | 18 | 19.93 (IQR: 24.37) | 15 | 0.580 |
| il10 | t3 | 35.92 (IQR: 36.18) | 24 | 34.28 (IQR: 37.52) | 18 | 0.638 |
| il10 | t4 | 19.27 (IQR: 45.99) | 23 | 13.27 (IQR: 39.29) | 24 | 0.444 |
| il10 | t5 | 25.6 (IQR: 54.67) | 23 | 48.61 (IQR: 69.31) | 19 | 0.841 |
| il10 | t6 | 27.9 (IQR: 36.06) | 13 | 5.76 (IQR: 112.69) | 6 | 0.759 |
| il1ra | t1 | 12.36 (IQR: 16.41) | 18 | 19.56 (IQR: 17.41) | 15 | 0.071 |
| il1ra | t3 | 8.67 (IQR: 19.88) | 24 | 16.68 (IQR: 35.4) | 18 | 0.137 |
| il1ra | t4 | 34.2 (IQR: 104.85) | 23 | 47.12 (IQR: 81) | 24 | 0.782 |
| il1ra | t5 | 154.1 (IQR: 195.12) | 23 | 190.3 (IQR: 280.51) | 19 | 0.870 |
| il1ra | t6 | 100.52 (IQR: 154.37) | 13 | 30.95 (IQR: 235.39) | 6 | 0.639 |
| il6 | t1 | 242.7 (IQR: 678.77) | 18 | 681.87 (IQR: 832.56) | 15 | 0.464 |
| il6 | t3 | 222.91 (IQR: 908.75) | 24 | 400.33 (IQR: 655.45) | 18 | 0.970 |
| il6 | t4 | 945.71 (IQR: 3042.11) | 23 | 591.08 (IQR: 980.87) | 24 | 0.276 |
| il6 | t5 | 1739.12 (IQR: 2015.1) | 23 | 847.08 (IQR: 1945.17) | 19 | 0.280 |
| il6 | t6 | 461.26 (IQR: 989.77) | 13 | 191.14 (IQR: 2906.58) | 6 | 0.701 |
| il8 | t1 | 444.94 (IQR: 1066.96) | 18 | 731.96 (IQR: 1071.71) | 15 | 0.845 |
| il8 | t3 | 363.36 (IQR: 438.6) | 24 | 471.67 (IQR: 443.63) | 18 | 0.950 |
| il8 | t4 | 922.16 (IQR: 2211.47) | 23 | 617.64 (IQR: 543.15) | 24 | 0.258 |
| il8 | t5 | 1041.63 (IQR: 876.22) | 23 | 1256.23 (IQR: 1707.52) | 19 | 0.452 |
| il8 | t6 | 1019.06 (IQR: 1313.03) | 13 | 1978.3 (IQR: 2268.84) | 6 | 0.368 |
| ip10 | t1 | 674.06 (IQR: 747.59) | 18 | 1256.9 (IQR: 1216.05) | 15 | 0.178 |
| ip10 | t3 | 691.37 (IQR: 873.32) | 24 | 901.34 (IQR: 1179.41) | 18 | 0.950 |
| ip10 | t4 | 2316.49 (IQR: 3508.53) | 23 | 2373.56 (IQR: 4323.83) | 24 | 1.000 |
| ip10 | t5 | 4809.84 (IQR: 7001.66) | 23 | 6174.1 (IQR: 7995.49) | 19 | 0.499 |
| ip10 | t6 | 5604.58 (IQR: 8923.48) | 13 | 1933.41 (IQR: 6754.7) | 6 | 0.701 |
| mcp1 | t1 | 8952.82 (IQR: 720.16) | 18 | 9254.12 (IQR: 4309.58) | 15 | 0.638 |
| mcp1 | t3 | 7647.28 (IQR: 6522.53) | 24 | 7808.71 (IQR: 6177.66) | 18 | 0.734 |
| mcp1 | t4 | 8796.58 (IQR: 6465.26) | 23 | 6395.84 (IQR: 7006.22) | 24 | 0.178 |
| mcp1 | t5 | 7841.98 (IQR: 6232.49) | 23 | 8103.67 (IQR: 6255.19) | 19 | 0.980 |
| mcp1 | t6 | 7758.99 (IQR: 5268.16) | 13 | 4852.7 (IQR: 6742) | 6 | 0.579 |
| mip1a | t1 | 9.05 (IQR: 9.44) | 18 | 20.09 (IQR: 13.69) | 15 | 0.158 |
| mip1a | t3 | 7.44 (IQR: 8.21) | 24 | 23.35 (IQR: 28.24) | 18 | 0.011 |
| mip1a | t4 | 22.25 (IQR: 26.7) | 23 | 40.38 (IQR: 40.53) | 24 | 0.177 |
| mip1a | t5 | 22.69 (IQR: 14.65) | 23 | 34.81 (IQR: 45.35) | 19 | 0.024 |
| mip1a | t6 | 16.36 (IQR: 32.22) | 13 | 48.51 (IQR: 23.33) | 6 | 0.179 |
| mip1b | t1 | 40.34 (IQR: 46.36) | 18 | 53.56 (IQR: 36.88) | 15 | 0.079 |
| mip1b | t3 | 25.2 (IQR: 22.52) | 24 | 56.6 (IQR: 34.59) | 18 | 0.020 |
| mip1b | t4 | 48.43 (IQR: 52.85) | 23 | 52.44 (IQR: 55.87) | 24 | 0.924 |
| mip1b | t5 | 27.79 (IQR: 25.22) | 23 | 40.3 (IQR: 67.67) | 19 | 0.032 |
| mip1b | t6 | 29.3 (IQR: 98.12) | 13 | 60.87 (IQR: 58.67) | 6 | 0.639 |
| pdgfaa | t1 | 47.4 (IQR: 56.94) | 18 | 106.23 (IQR: 106.02) | 15 | 0.033 |
| pdgfaa | t3 | 34.4 (IQR: 40.41) | 24 | 54.59 (IQR: 62.77) | 18 | 0.489 |
| pdgfaa | t4 | 14.79 (IQR: 20.99) | 23 | 20.86 (IQR: 10.97) | 24 | 0.381 |
| pdgfaa | t5 | 13.67 (IQR: 6.33) | 23 | 18.97 (IQR: 17.01) | 19 | 0.452 |
| pdgfaa | t6 | 16.79 (IQR: 9.54) | 13 | 14.09 (IQR: 4.15) | 6 | 0.629 |
| pdgfabbb | t1 | 21.47 (IQR: 43.48) | 18 | 40.51 (IQR: 27.14) | 15 | 0.262 |
| pdgfabbb | t3 | 24.75 (IQR: 28.53) | 24 | 26.38 (IQR: 29.05) | 18 | 0.859 |
| pdgfabbb | t4 | 13.72 (IQR: 13.08) | 23 | 11.99 (IQR: 10.1) | 24 | 0.733 |
| pdgfabbb | t5 | 14.34 (IQR: 15.1) | 23 | 15.91 (IQR: 7.57) | 19 | 0.879 |
| pdgfabbb | t6 | 10.8 (IQR: 3.12) | 13 | 12.86 (IQR: 5.03) | 6 | 0.271 |
| rantes | t1 | 50.45 (IQR: 165.18) | 18 | 199.13 (IQR: 297.78) | 15 | 0.430 |
| rantes | t3 | 2.23 (IQR: 0) | 24 | 2.23 (IQR: 0) | 18 | 0.986 |
| rantes | t4 | 7.41 (IQR: 13.8) | 23 | 7.68 (IQR: 11.19) | 24 | 0.983 |
| rantes | t5 | 4.48 (IQR: 6.67) | 23 | 5.88 (IQR: 3.91) | 19 | 0.676 |
| rantes | t6 | 3.61 (IQR: 2.64) | 13 | 4.48 (IQR: 2.37) | 6 | 0.265 |
| scd40l | t1 | 4.63 (IQR: 2.72) | 18 | 6.14 (IQR: 11.02) | 15 | 0.372 |
| scd40l | t3 | 4.63 (IQR: 4.9) | 24 | 4.63 (IQR: 7.95) | 18 | 0.663 |
| scd40l | t4 | 3.8 (IQR: 5.59) | 23 | 3.8 (IQR: 6.56) | 24 | 0.784 |
| scd40l | t5 | 11.9 (IQR: 6.93) | 23 | 10.65 (IQR: 8.55) | 19 | 0.771 |
| scd40l | t6 | 4.08 (IQR: 8.12) | 13 | 8.26 (IQR: 8.5) | 6 | 0.658 |
| tnfa | t1 | 1.22 (IQR: 2.1) | 18 | 1.97 (IQR: 1.84) | 15 | 0.489 |
| tnfa | t3 | 1.18 (IQR: 1.15) | 24 | 1.88 (IQR: 5.67) | 18 | 0.051 |
| tnfa | t4 | 4.46 (IQR: 3.43) | 23 | 4.76 (IQR: 4.28) | 24 | 0.873 |
| tnfa | t5 | 5.88 (IQR: 4.25) | 23 | 9.62 (IQR: 11.63) | 19 | 0.046 |
| tnfa | t6 | 8.31 (IQR: 13.27) | 13 | 11.25 (IQR: 13.59) | 6 | 0.965 |

* within 24 hours of admission (T1), 24–48 hours (T3), days 3–5 (T4), days 6–8 (T5), over 8 days but prior to discharge (T6), and at 30 (TM30), 60 (TM60), and 90 (TM90) days post-ictus
